# PRC1 nanoglobules organize *Hox* chromatin during *Drosophila* embryogenesis

**DOI:** 10.1038/s41421-026-00902-8

**Published:** 2026-07-07

**Authors:** Thierry Cheutin, Nazli Akilli, Marco Di Stefano, Paul-Swann Puel, Lauriane Fritsch, Daniel Jost, Giacomo Cavalli

**Affiliations:** 1 Institute of Human Genetics, University of Montpellier, CNRS, Montpellier, France; 2 Laboratoire de Biologie et Modélisation de la Cellule, École Normale Supérieure de Lyon, CNRS, UMR5239, Inserm U1293, Université Claude Bernard Lyon 1, Lyon, France

**Keywords:** Chromatin, Gene silencing

## Abstract

Polycomb group (PcG) proteins are required to maintain the silencing of key developmental genes during development. In *Drosophil*a, they bind to discrete genomic elements named Polycomb response elements (PREs). PcG proteins form two classes of complexes. Polycomb repressive complex 2 (PRC2) deposits H3K27me3, a histone mark that covers large chromatin domains. The canonical PRC1 complex can bind to PREs as well as to the H3K27me3 mark. Inside the cell nucleus, when analyzed by confocal microscopy, the PcG subunits and associated chromatin are localized to polycomb foci. However, the lack of spatial resolution of this method impedes the precise localization of the polycomb machinery relative to its target chromatin. To resolve this long-standing problem, we analyzed *Drosophila* embryos by superresolution stimulated emission depletion microscopy. We observed that polycomb foci associated with *Hox* gene clusters are composed of multiple dynamic PRC1 substructures, which we named “PRC1 nanoglobules”. PREs are more associated with these nanoglobules than the surrounding H3K27me3 chromatin. By imaging the entire Bithorax complex (BX-C) locus, we identified chromatin substructures that partially overlap with PRC1 nanoglobules, indicating that they form different assemblies. Furthermore, polymer simulations suggest that interactions between PREs and PRC1 nanoglobules can drive the compaction and positioning of the BX-C domain. Taken together, these data suggest that by establishing the three-dimensional architecture of their target loci, PRC1 nanoglobules are key molecular assemblies that maintain polycomb-dependent silencing.

## Introduction

Several regulatory layers control gene expression during cell differentiation. While transcription factors act on discrete elements such as promoters and enhancers to induce transcription^1,2^, polycomb components are able to maintain the silencing of most developmental genes outside their normal spatiotemporal expression domains^3^. Polycomb group (PcG) proteins belong mainly to two types of polycomb repressive complexes (PRCs), both of which are required for gene silencing. PRC2 catalyzes the methylation of H3K27, whereas PRC1 can ubiquitylate H2AK119 (H2AK118 in flies), and each of the two groups of complexes can reinforce silencing through their association with the histone mark deposited by the other group^4,5^. PcG proteins bind to specific sites, called polycomb response elements (PREs), in *Drosophila*^3^. PRC1 forms several variant complexes depending on the presence of alternative subunits: so-called canonical PRC1 maintains gene silencing, whereas binding of noncanonical PRC1 starts direct repression of transcription, notably by recruiting PRC2^6^. Once recruited, PRC2 deposits H3K27me3 in the chromatin surrounding PREs, generating chromatin domains coated with H3K27me3. This mark is then recognized by canonical PRC1, which, in flies, contains the polyhomeotic (Ph) and polycomb (Pc) subunits. Transgenesis experiments have shown that PREs are DNA sequences that are necessary and sufficient for the recruitment of PcG subunits and the silencing of their flanking genes^3,7^. However, studies attempting to predict the genomic localization of PcG proteins and analyses of endogenous deletions of PREs have provided a complex picture, emphasizing the role of neighboring chromatin in determining the actual binding of PcG proteins to PREs^8–10^. Furthermore, H3K27me3, the histone mark deposited by PRC2, spreads across large chromatin domains, strongly suggesting that polycomb function is linked to the higher-order organization of the linear genome^11–13^. In the three-dimensional (3D) nuclear space, PRC1 subunits accumulate in structures called polycomb foci, which have been shown to colocalize with repressed chromatin covered with H3K27me3^14–16^. Additionally, long-range interactions between H3K27me3 chromatin domains involve polycomb foci^17–20^. Although there is evidence that polycomb-dependent silent chromatin is more compacted than the same genomic regions in cells in which the corresponding genes are expressed^18,21^, the mechanism of compaction is more controversial^22,23^. Indeed, the effect of PcG proteins on chromatin compaction could be direct or indirect, as the release of gene repression could also lead to chromatin decompaction. We previously demonstrated that the deletion of PRC1 subunits affects homeobox (*Hox*) cluster chromatin compaction prior to any ectopic *Hox* gene expression during early *Drosophila* embryogenesis, suggesting that PRC1 directly condenses *Hox* clusters^21^. However, how the mild effect of PRC1 on *Hox* cluster compaction maintains *Hox* gene silencing, as well as the PRC1-induced compaction mechanism, remain enigmatic. Recent studies have reported that some canonical PRC1 subunits can make liquid-like droplets in vitro, suggesting that polycomb foci correspond to condensates formed by phase separation^24–29^. Interestingly, this biochemical property would explain the formation of higher-order structures and suggests that polycomb function does not rely solely on the direct binding of its components to chromatin but may be driven by self-association properties. However, the lack of optical microscopy resolution has hampered the fine characterization of these nuclear structures in the past, making it impossible to link the linear distribution of PcG proteins along the genome with their precise 3D localization relative to the associated chromatin.

The polycomb machinery acts during cell differentiation when chromatin is extensively reorganized by modifications of histone marks and changes in chromatin 3D organization^8,30^. Here, we analyzed the higher-order organization of canonical PRC1 components in the nuclear space in relation to two of their best characterized *Drosophila* target loci, the BX-C and Antennapedia complex (ANT-C), clusters of *Hox* genes^31,32^. These two gene clusters host the two largest H3K27me3 domains, contain many PREs and appear under microscopy as the most intense polycomb foci in the cell nuclei of *Drosophila* embryos^21,32^. Interestingly, H2AK118Ub was shown to be dispensable for the function of polycomb on *Hox* genes^33^, which allows one to focus specifically on the relationship between H3K27me3 and polycomb components when these loci are being studied. A further advantage is the large size of both clusters (over 300 kb each), which reduces the challenge posed by the limited spatial resolution that characterizes even superresolution microscopy methods. Here, we used stimulated emission depletion (STED) microscopy to study the 3D nuclear localization of Ph and Pc relative to repressed *Hox* chromatin in *Drosophila* embryos. Our data show that the largest polycomb foci are composed of multiple substructures approximately 70 nm in diameter, which we named “PRC1 nanoglobules”. We confirmed their presence in living embryos using an orthogonal superresolution microscopy method called AiryScan microscopy and showed that PRC1 nanoglobules move rapidly and reshape constantly. Immunofluorescence in situ hybridization (immuno-FISH) experiments revealed that PREs are more often associated with PRC1 nanoglobules than the remaining H3K27me3-coated *Hox* chromatin. Importantly, through biophysical chromatin modeling using polymer simulations, we linked the genomic position of PcG proteins and H3K27me3 to the localization of polycomb target chromatin relative to PRC1 nanoglobules and predicted Ph-dependent compaction of the BX-C locus. Taken together, the results of this study demonstrate that PRC1 subunits form higher-order structures that fold polycomb-associated chromatin.

## Results

### Intense polycomb foci are composed of multiple PRC1 nanoglobules

**Fig. 1:**
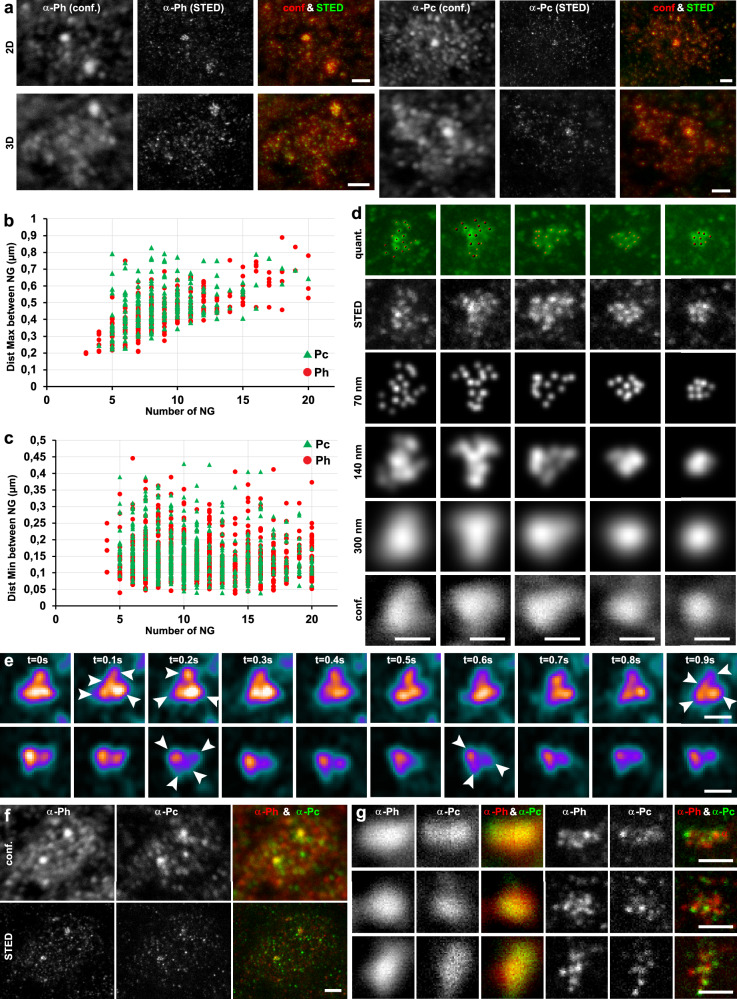
Large polycomb foci are composed of several PRC1 nanoglobules. **a** Two examples of confocal, STED, and merged Ph and Pc immunostaining images of the head of *Drosophila* embryos. A single optical section is shown in 2D images, whereas the 3D view corresponds to a projection of 5 sections. The bars measure 1 µm. **b** Scatterplot showing the average maximum distance between nanoglobules of one focus and its number of nanoglobules for both Ph and Pc foci. **c** Scatterplot showing the number of nanoglobules and the minimum distance between nanoglobules of one focus for both Ph and Pc foci. **d** Five examples illustrating the effect of resolution on images of large polycomb foci. Centers of nanoglobules identified by processing STED images (first row) can be used to compute images using an artificial p.s.f. of 70 (same resolution as the STED images), 140 (same resolution as the AiryScan images), and 300 nm (same resolution as the confocal images). The bars represent 500 nm. **e** Two time-lapse imaging showing one picture every 0.1 s of large Ph foci imaged in the head of *Drosophila* embryos expressing Ph-GFP with AiryScan microscopy. The shape and internal organization of large Ph foci change rapidly over time, indicating that they are composed of several mobile substructures (arrowheads). The bars represent 500 nm. **f** Confocal and STED images of a cell nucleus located in the head of a *Drosophila* embryo illustrating the colocalization of Ph and Pc. The bar represents 1 µm. **g** Three examples showing the colocalization of Ph and Pc in large polycomb foci imaged in confocal and STED microscopies. The bars represent 500 nm.

The observation of large H3K27me3 genomic domains and the ability of several PRC1 subunits to phase separate suggest that the organization of PRC1 in the 3D nuclear space is essential for PRC1 function. In particular, the Ph subunit of the *Drosophila* PRC1 complex has been shown to form large condensates in vitro^24–28^, suggesting that it may play a crucial role in the formation of nuclear foci. To test this hypothesis, we generated *Drosophila* lines in which a GFP tag was added to the endogenous *Psc*, *Sce* or *Pc* genes by CRISPR-Cas9 knock-in, and we imaged the nuclear distribution of Psc-GFP, Sce-GFP and Pc-GFP in ph null mutant embryos. As expected, the accumulation of the three PRC1 subunits within nuclear foci was greatly reduced in mutant embryos (Supplementary Fig. S1a), demonstrating that the Ph subunit is necessary for the formation of PRC1 foci. To further investigate the ability of PRC1 proteins to form large, liquid-like condensates inside the cell nucleus, we imaged *Drosophila* embryos using superresolution microscopy. If the large foci observed by confocal microscopy correspond to standard liquid-like droplets, STED microscopy should reveal large foci that should not separate into disconnected substructures. Instead, Ph or Pc immunostaining imaged by STED microscopy revealed that many polycomb foci are composed of substructures ~70 nm in diameter and characterized by a heterogeneous signal intensity, which suggests that they contain a variable number of PcG molecules. We named them “PRC1 nanoglobules” (Fig. 1a). To describe their distribution within the cell nucleus, we defined clusters of nanoglobules as sets of signals that were less than 140 nm apart. While the majority (approximately 70%) of the nanoglobules were isolated, the others were grouped into clusters of several nanoglobules (Supplementary Fig. S1b).

To characterize the internal structure of the most intense polycomb foci, we quantified the STED signals contained within the areas previously defined by the segmentation of large polycomb foci in confocal images (Supplementary Fig. S1c). In the heads of embryos, where all *Hox* genes are repressed, the most intense polycomb foci, which generally correspond to the largest polycomb domains in the genome, namely, the BX-C and ANT-C *Hox* clusters^15^, showed high morphological variability, ranging from 4 to 20 nanoglobules separated by a maximum distance between them of 200 to 800 nm (Fig. 1b). Interestingly, the minimum distance between nearest-neighbor substructures was most often less than 200 nm, i.e., lower than the spatial resolution of confocal microscopy, which explains why superresolution is required to distinguish them (Fig. 1c). To model the effect of spatial resolution on the visualization of large polycomb foci, we used the coordinates of each nanoglobule identified after the quantification of the STED images and artificially reconstructed the corresponding images that such substructures would produce when using simulated point spread functions (p.s.f.) of 70, 140, and 300 nm (Fig. 1d). Modeling using a p.s.f. of 70 nm produced virtual images that well recapitulated experimental STED images, whereas a p.s.f. of 300 nm mimicked confocal images. Interestingly, an intermediate resolution of 140 nm could still capture some substructures within polycomb foci (Fig. 1d), suggesting that imaging at an intermediate resolution would be sufficient to identify the composite nature of large polycomb foci. To exploit this feature and track the dynamics of polycomb foci in vivo, we generated a *Drosophila* line in which a GFP tag was added to the endogenous *ph-d* gene (one of the two paralogous genes coding for the Ph subunit of PRC1) by CRISPR-Cas9 knock-in. Timelapse AiryScan microscopy experiments on live embryos revealed that intense polycomb foci do not form homogenous and immobile structures. Instead, they are composed of several substructures moving rapidly relative to one another (Fig. 1e). Individual substructures could be observed in each frame of the timelapse experiment, suggesting the presence of fusion and demixing events between them. Importantly, the observation of substructures by live microscopy ruled out that PRC1 nanoglobules are artifacts due to the formaldehyde fixation or the immunostaining process, nor are they microscopic aberrations due to the procedure of STED imaging.

We observed nanoglobules inside the most intense foci for multiple subunits of PRC1, namely, Ph, Pc and Psc (Supplementary Fig. S1d). To test whether different PRC1 subunits colocalize within these nanostructures, we performed two-color immunostaining to detect both the Ph and Pc subunits by STED microscopy upon imaging of embryo heads. As previously shown^15^, the two proteins were found to colocalize by confocal microscopy (Fig. 1f, g; Supplementary Fig. S1e). Similarly, the Ph and Pc nanostructures showed a high degree of overlap by STED microscopy (Fig. 1f, g; Supplementary Fig. S1e), indicating that the intense polycomb foci are composed of nanoglobules containing multiple subunits of PRC1. Taken together, these observations demonstrate that intense polycomb foci are composed of multiple PRC1 nanoglobules in *Drosophila* embryos.

### PRC1 nanoglobules associate more frequently with PREs than with intermediate regions

**Fig. 2:**
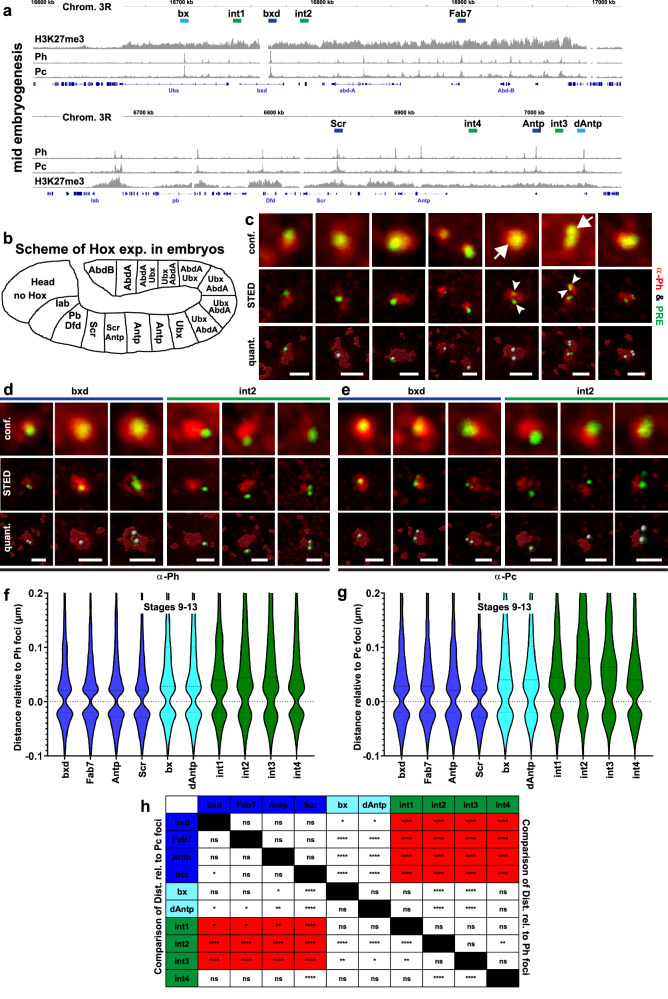
Compared with intermediate regions, PREs interact more with Ph/Pc substructures. **a** Genomic maps showing the H3K27me3, Ph and Pc profiles observed during mid-embryogenesis^37^ and the localization of FISH probes in BX-C and ANT-C. PRE probes are shown in blue, whereas probes for intermediate regions are shown in green. **b** Scheme of a fly embryo representing the pattern of *Hox* gene expression along the antero-posterior axis. **c** Confocal, STED, and segmented (quant.) images of a PRE (green) compared with Ph foci (red) acquired in the head of *Drosophila* embryos. STED images show that a single FISH signal (arrow) acquired by confocal microscopy can be composed of two spots (arrowheads). Moreover, STED images of immunolabeling can be robustly segmented (red objects in quant. pictures). The bars represent 500 nm. **d**, **e** Confocal, STED, and segmented (quant.) images illustrating the localization of one PRE (bxd) or one intermediate region (int2) relative to Ph (**d**) or Pc (**e**) immunolabeling. STED and segmented images show that int2 is less associated with Ph or Pc immunolabeling. The bars measure 500 nm. **f**, **g** Violin plots showing the distribution of minimum distances measured between the center of FISH spots and the border of Ph (**f**) or Pc (**g**) substructures in embryos from stages 9 to 13 (mid-embryogenesis). Distances are negative when FISH spots are inside Ph (or Pc) substructures and positive when they are outside. With the exception of bx and dAntp (light blue), which have an intermediate distribution, PREs located in the middle of the *Hox* clusters (dark blue) are more associated with Ph or Pc substructures than intermediate regions (green). **h** Table of statistical significance from the pairwise comparisons of the distance distributions shown in panels **f** (Ph: upper triangle of the matrix) and **g** (Pc: lower triangle of the matrix). Relative to Ph substructures, PREs located in the middle of *Hox* clusters (dark blue) and intermediate regions (green) form two groups with different distance distributions (red). Compared with Pc substructures, intermediate regions show more variation. Nevertheless, with the exception of int4, the 3 other intermediate regions are significantly less associated (red) with Pc staining than are the PREs located in the middle of the *Hox* clusters. ns, not significant; *P < 0.05; **P < 0.01; ***P < 0.001; ****P < 0.0001.

In *Drosophila*, chromatin immunoprecipitation (ChIP) experiments showed that polycomb-associated chromatin is composed of discrete PREs, where PRC1 and PRC2 proteins bind strongly, flanked by large regions covered with H3K27me3 with weaker PcG protein binding^11,12^, which we call “intermediate regions” in this study. However, the 3D organization of PREs and flanking regions relative to the nuclear localization of polycomb complexes is unknown. To test whether these two types of chromatin regions have a different localization with respect to PRC1 nanoglobules, we designed ten 6 kb probes targeting 6 PREs (3 in BX-C and 3 in ANT-C) and 4 intermediate regions (Fig. 2a and Supplementary Table S1). We used them to perform Ph or Pc immuno-FISH experiments with *Drosophila* embryos and examine the embryo head (Fig. 2b). The observation of 2 or 3 FISH spots (Fig. 2c) and rarely 4 (Supplementary Fig. S5b) associated with a single polycomb focus suggested that paired homologous chromosomes and sister chromatids can be distinguished by STED microscopy. To quantify the localization of PREs and intermediate regions relative to Ph- or Pc-immunolabeled regions, we calculated the minimum distance between the center of FISH spots and the boundary of Ph or Pc substructures: distances are negative when the FISH spot centers were inside immunolabeled structures and positive when they were outside. PREs located in the middle of *Hox* clusters were significantly more associated with Ph nanostructures than intermediate regions (Fig. 2d, f, h). Similar results were observed with Pc nanostructures, with the exception of the intermediate region int4, for which the data indicate a lower interaction frequency, but the difference was not significant (Fig. 2e, g, h). Moreover, bx and dAntp, two PREs located near the H3K27me3 domain boundary, exhibited an intermediate distribution (Fig. 2f–h), indicating that the PRE position in *Hox* clusters might also affect their localization relative to PRC1 nanoglobules.

To confirm that the association of intermediate regions with PRC1 nanoglobules is not a consequence of their genomic proximity to PREs, we designed three additional probes located in repressed chromatin adjacent to *Hox* clusters and separated from H3K27me3 domains with a genomic distance comparable to those between intermediate regions and their closest PREs (Supplementary Fig. S2a). These three probes located outside *Hox* clusters were significantly less associated with Ph or Pc substructures than intermediate regions (Supplementary Fig. S2b–g), demonstrating that PRC1 nanoglobules specifically shape intermediate regions. Notably, the adjacent probe e-AbdB, where H3K27me3 extends slightly (see the arrow in Supplementary Fig. S2a), has a distance distribution closer to intermediate regions than the other two adjacent probes, which are not covered by H3K27me3 (Supplementary Fig. S2b–g). In addition, immuno-FISH experiments performed with probes designed into repressed chromatin regions not enriched in specific repressive histone marks (void chromatin) and located away from H3K27me3 domains revealed that this chromatin type is even less associated with Ph substructures than loci adjacent to *Hox* clusters are (Supplementary Fig. S2b, d, f).

Finally, we analyzed the effect of transcription on the localization of polycomb-associated chromatin relative to that of Ph or Pc nuclear foci by performing immuno-FISH experiments in parasegments where the corresponding *Hox* genes are expressed (see scheme in Fig. 2b). Consistent with previous studies reporting that polycomb foci contain only repressed chromatin^21,34^, transcription of the *Hox* gene regulated by each of the PREs strongly decreased the association of PREs and of the relative intermediate regions with Ph or Pc substructures (Supplementary Fig. S3). Taken together, these results demonstrate that PRC1 nanoglobules colocalize with repressed *Hox* chromatin. However, these associations are probabilistic because PREs contact Ph/Pc substructures in only ~60% of cell nuclei, a frequency that decreases only to ~50% for intermediate regions. This work highlights the interest in using single-cell approaches because they show that interactions of small genomic regions with PRC1 nanoglobules occur in a cell subpopulation at any given time. Most likely, given the rapid movement observed by real-time microscopy, this observation indicates that the interaction between PRC1 nanoglobules and their target chromatin occurs in a transient manner.

### Deletion of the Pc subunit affects the association of PREs and intermediate regions with PRC1 nanoglobules

**Fig. 3:**
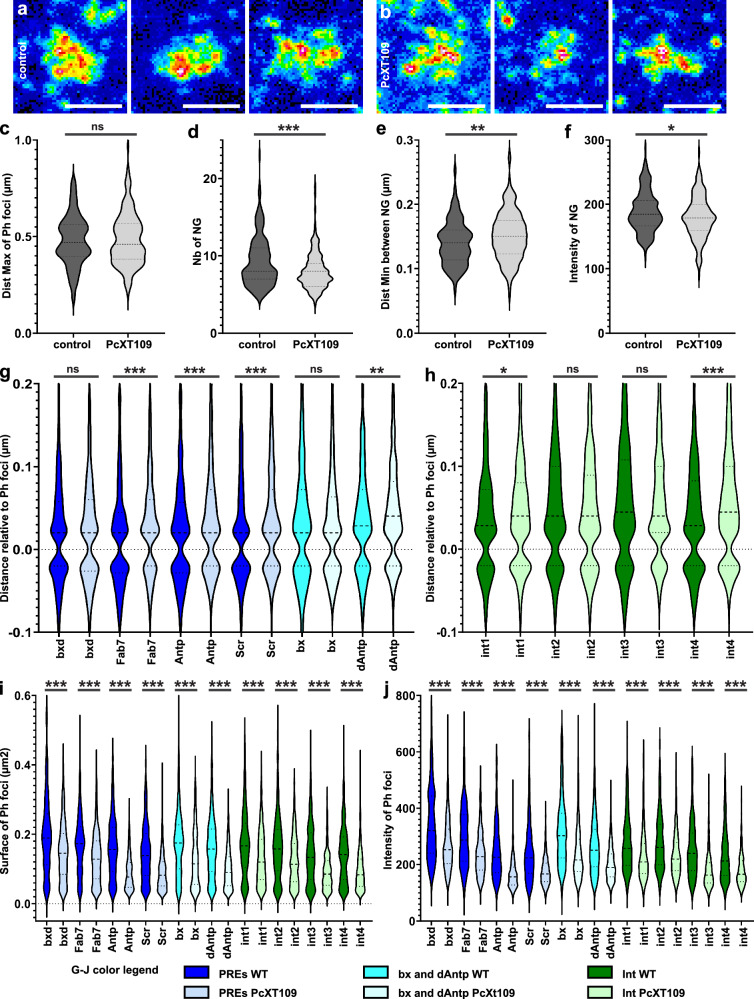
Effect of Pc on Ph nanoglobules and their association with *Hox* chromatin. **a**, **b** Three examples of large Ph foci imaged in the head of control (**a**) or PcXT109 (**b**) embryos using STED microscopy. The bars represent 0.5 µm. **c**–**f** Comparison of large Ph foci imaged in the heads of control (dark gray) and PcXT109 (null mutant for Pc) embryos (light gray). Violin plots compare the average maximum distance between nanoglobules of one Ph focus (**c**), the number of nanoglobules within one Ph focus (**d**), the average minimum distance between nanoglobules of one Ph focus (**e**) and the intensity of immunolabeling in nanoglobules within one Ph focus (**f**). **g**, **h** Violin plots comparing the distribution of minimum distances measured between the center of FISH spots and the border of Ph substructures in control and PcXT109 embryos. In the heads of the embryos, some PREs are less associated with Ph substructures in the Pc mutant embryos, whereas bxd and bx are not affected (**g**). Similarly, Pc does not affect the localization of all intermediate regions, since only int1 and int4 are less associated with substructures in Pc-mutant embryos (**h**). **i**, **j** Violin plots comparing the surface (**i**) and intensity (**j**) of the Ph substructures located less than 100 nm from FISH spots in control and PcXT109 embryos. ns, not significant; *P < 0.05; **P < 0.01; ***P < 0.001.

In a previous study, we used confocal microscopy to show that the Ph subunit still accumulates within nuclear foci in the absence of the Pc subunit of PRC1^21^. Moreover, the effect on the compaction of chromatin associated with repressed *Hox* clusters was weaker in Pc mutant embryos than in Ph mutant embryos, suggesting that the Ph subunit can still partially compact *Hox* clusters in the absence of the Pc subunit. To test the effect of loss of the Pc subunit on the formation of Ph nanoglobules, we imaged Ph immunostaining in Pc null mutant embryos by superresolution STED microscopy. We found that the number of nanoglobules within the largest Ph foci decreased and the minimum distance between them increased in the absence of Pc, whereas the overall size of Ph foci did not change (Fig. 3a–f). Since Pc is the only PRC1 subunit capable of binding H3K27me3^3^, one hypothesis is that the function of Pc within PRC1 might be to favor the transient association of PRC1 with H3K27me3-coated regions to reinforce silencing. Alternatively, Pc might also help PRC1 bind to PREs. Immuno-FISH experiments followed by STED imaging revealed that the localization of the bxd and bx PREs relative to Ph nanoglobules was not affected by the absence of the Pc subunit, whereas the other PREs decreased their association with Ph substructures (Fig. 3g). Notably, the enrichment of the Ph subunit on bxd and bx, as measured by chromatin immunoprecipitation-sequencing (ChIP-seq), was greater than that on Fab7 or other PREs of the ANT-C locus (Fig. 2a). The strong affinity of Ph for these target sites might explain why the localization of bx and bxd is preserved in the absence of the Pc subunit. Similar to those of PREs, the associations of some intermediate regions with Ph foci are not decreased in Pc mutant embryos, whereas other intermediate regions show weaker associations with Ph substructures in the absence of the Pc subunit (Fig. 3h). The comparable effect on PREs and intermediate regions observed in Pc mutant embryos demonstrates that Pc is not absolutely required but favors the association of both types of chromatin regions with Ph substructures. Indeed, compared with those of control embryos, the area and intensity of Ph foci located within 100 nm of the FISH signal in Pc mutant embryos decreased for all probes tested (Fig. 3i, j), indicating that the absence of Pc induces a global effect, weakening Ph substructures.

### *Hox* chromatin preferentially localizes to the periphery of PRC1 nanoglobules during embryogenesis

**Fig. 4:**
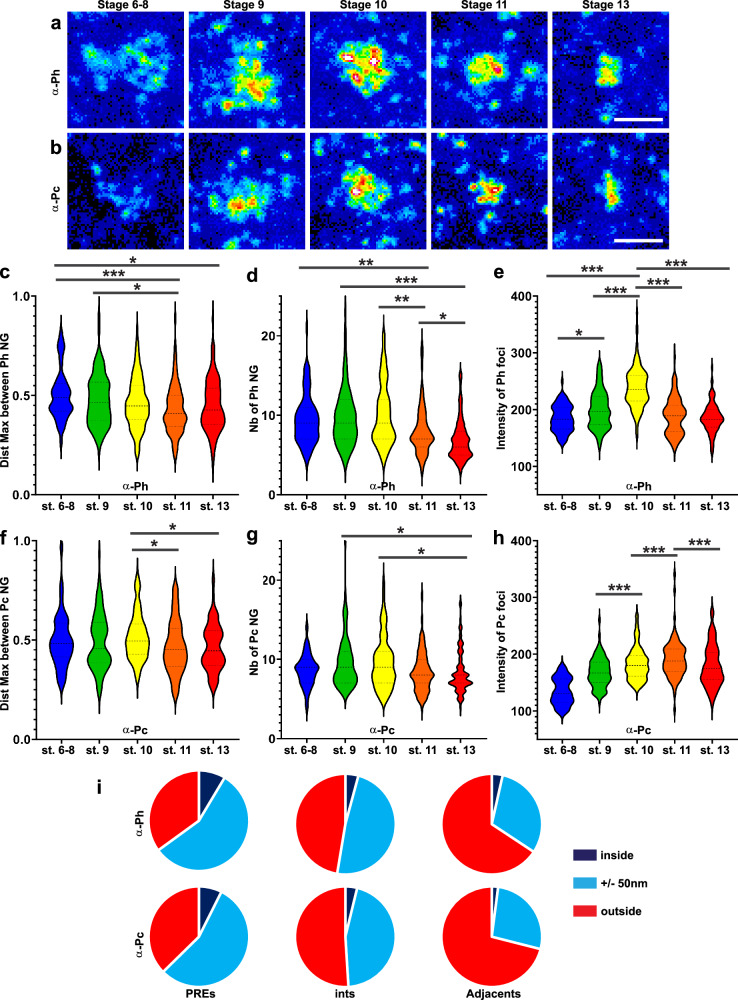
Modification of large PRC1 foci during embryogenesis. **a**, **b** STED images illustrating the structural changes of large Ph (**a**) or Pc (**b**) foci in the heads of embryos at stages 6–8, 9, 10, 11, and 13. The bars represent 0.5 µm. **c**–**h** Quantification of large Ph (**c**–**e**) or Pc (**f**–**h**) foci during embryogenesis. Violin plots compare the average maximum distance between nanoglobules (**c**, **f**), the number of nanoglobules (**d**, **g**), and the intensity of immunolabeling in nanoglobules within one focus (**e**, **h**) in the head of *Drosophila* embryos. **i** Pie charts of the frequency of FISH spots localized inside, near the boundary, and outside the Ph/Pc substructures in stages 9–13. Both the PREs and intermediate regions are often located close to the boundary (± 50 nm) of the Ph/Pc substructures.

Polycomb foci form during early embryogenesis through the progressive accumulation of PRC1 subunits on polycomb-targeted chromatin. Concomitantly, we previously showed that the residence time of the proteins forming these foci increases, which is consistent with stronger binding of PRC1 subunits to polycomb foci^15^. In addition, the stability of polycomb-dependent repression increases during development, since the inhibition of a transgene by the polycomb machinery can be irreversibly challenged during embryogenesis but not during larval stages^35^. These changes associated with polycomb biology during development prompted us to study the structural dynamics of PRC1 nanoglobules during embryogenesis. We first used AiryScan microscopy to analyze a *Drosophila* line expressing Ph-GFP. Ph substructures can be observed at all embryonic stages, but the largest Ph foci exhibit larger, more numerous, and more mobile substructures during early embryogenesis (stages 5–10) than during stages 11–13 (Supplementary Fig. S4). Consistently, imaging of Ph or Pc immunostaining by STED microscopy revealed that the maximum distance between PRC1 nanoglobules and their number in large Ph (Fig. 4a, c–e) or Pc (Fig. 4b, f–h) foci decreased during embryogenesis, whereas the intensity of Ph or Pc immunostaining in nanoglobules increased until mid-embryogenesis (Fig. 4e, h). An important question is what effect this progressive organization has on the spatial organization of *Hox* genes. Therefore, we performed immuno-FISH experiments on the heads of embryos at different embryonic stages (representative images are shown for the Fab7 DNA FISH probe in Supplementary Fig. S5). First, we compared the localization of PREs and intermediate regions by combining the distance distributions for PREs (bxd and Fab7 for BX-C; Scr and Antp for ANT-C) and for intermediate regions (int1 and 2 for BX-C; int3 and 4 for ANT-C). Except during the formation of Ph/Pc foci at embryonic stages 5 to 8, PREs were always significantly more associated with Ph or Pc substructures than intermediate regions during later embryonic stages (Supplementary Fig. S6a–d). Second, we focused on changes in chromatin association with PRC1 nanoglobules during embryogenesis. From embryonic stages 5 to 8, the association of BX-C and ANT-C chromatin with Ph or Pc substructures increased (Supplementary Fig. S6e–h), which is consistent with the accumulation of both Ph and Pc in polycomb foci during early embryogenesis (see Supplementary Fig. S5). During later embryogenesis, the distance distributions between the BX-C and Ph/Pc substructures remained similar, and probes located in ANT-C were only slightly less associated with Ph/Pc foci at stage 13 (Supplementary Fig. S6g, h). Overall, while the shape of Ph/Pc large polycomb foci changed from stage 9 to stage 13, the position of polycomb-associated chromatin relative to that of PRC1 nanoglobules was quite stable. Furthermore, the PREs and intermediate regions of BX-C and ANT-C were enriched near the boundary ( ± 50 nm) of the Ph/Pc substructures (Fig. 4i), suggesting that their periphery is the main location of interaction between polycomb-associated chromatin and PRC1 higher-order structures.

### BX-C chromatin forms substructures different from those of PRC1 nanoglobules

**Fig. 5:**
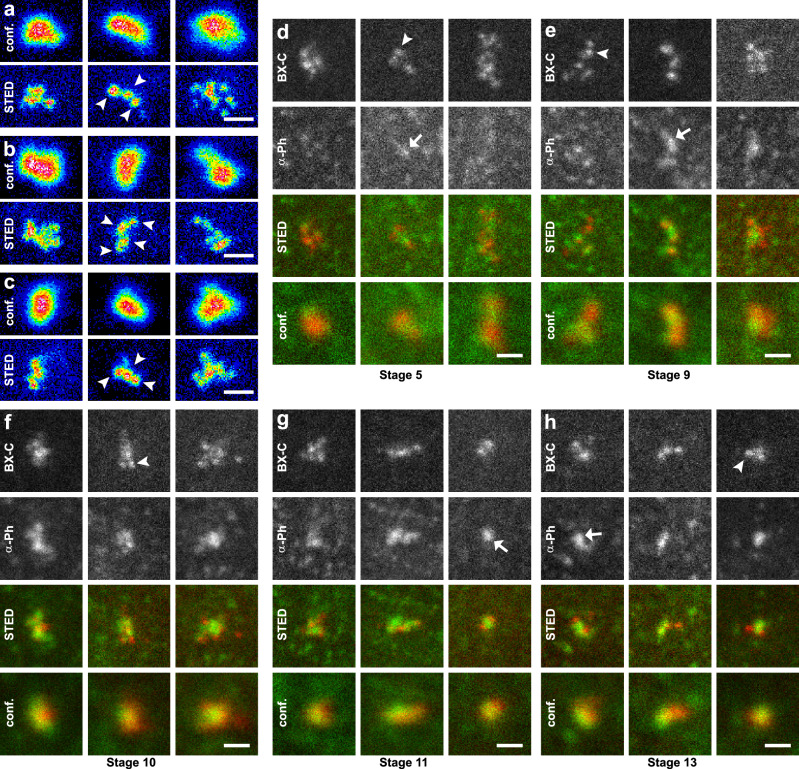
Whole BX-C forms chromatin nanoglobules partially overlapping with Ph nanoglobules. **a**–**c** Whole BX-C imaged by confocal and STED microscopies in the head of *Drosophila* embryos at stages 5 (**a**), 10 (**b**), and 13 (**c**). Although confocal microscopy revealed a homogenous distribution of BX-C, STED imaging revealed infrastructures that we named chromatin nanoglobules (arrowheads). The bars represent 500 nm. **d**–**h** Pictures of whole BX-C and Ph immunolabeling imaged in the head of *Drosophila* embryos at stages 5 (**d**), 9 (**e**), 10 (**f**), 11 (**g**), and 13 (**h**). Some BX-C chromatin nanoglobules are formed without Ph nanoglobules (arrowheads), and Ph nanoglobules are often seen connecting BX-C chromatin nanoglobules (arrows), indicating that chromatin nanoglobules and Ph nanoglobules form different structures. The bars represent 500 nm.

Given the data shown above, it is clear that despite being fully repressed by polycomb components and coated by H3K27me3, the chromatin of *Hox* loci is not entirely and always contained within polycomb condensate structures. We therefore wished to establish the spatial relationship between the ChIP-seq binding patterns of PRC1 and the shape of the whole locus. The BX-C locus is characterized by approximately twenty PRC1 peaks distributed over a region of more than 300 kb (Fig. 2a). Several structural scenarios might correspond to these ChIP-seq data. One possibility is that peaks might form a backbone, with their corresponding DNA generally associated with (and thus coinciding with) PRC1 nanoglobules and with the intervening regions surrounding them. Alternatively, PRC1 proteins might form structures that associate but are not coincident with DNA signals if multiple PRC1 complexes associate and only a fraction of them bind directly to DNA. We therefore used an oligopaint probe covering the entire BX-C domain to perform FISH experiments on the heads of *Drosophila* embryos. Since there is no transcription in fly embryo heads throughout the region and since this structure forms a homogeneous domain in Hi-C experiments from mid to late embryogenesis^8^, it might have been expected that this domain would fold into a homogeneous globular structure, but the results indicate a different picture. While the structure of the BX-C locus appears fairly homogeneous by confocal microscopy, STED microscopy revealed multiple substructures, indicating that BX-C chromatin forms several “chromatin nanoglobules”. Consistent with the previously described compaction of BX-C during embryogenesis^21^, chromatin nanoglobules appeared more dispersed in stage 5, whereas their arrangements were more difficult to resolve during later embryogenesis (Fig. 5a–c). To test whether these BX-C chromatin substructures corresponded to Ph nanoglobules, we performed immuno-FISH followed by STED microscopy. Regardless of the embryonic stage, BX-C and Ph nanoglobules formed closely associated but different assemblies that only partially overlapped. Moreover, BX-C nanoglobules could be observed without Ph nanoglobules (arrowheads in Fig. 5d–h), and PRC1 nanoglobules were often observed connecting BX-C nanoglobules (arrows in Fig. 5d–h). Taken together, these results suggest that PRC1 nanoglobules are dispensable for the formation of BX-C chromatin nanoglobules but might lead to the 3D organization of PREs and intermediate regions near their surface.

To verify that these observations were not specific to the BX-C locus, we performed additional immuno-FISH experiments with oligopaint probes covering single or multiple H3K27me3 domains^36^. Consistent with the genomic data showing that these domains correspond to polycomb-associated chromatin (called blue chromatin in Supplementary Fig. S7a, b), they were associated with PRC1 nuclear substructures (arrowheads in Supplementary Fig. S7a, b). However, the Ph and chromatin substructures are not identical since their overlap was only partial (arrows in Supplementary Fig. S7a, b), similar to the observations for BX-C chromatin. To confirm that the association of chromatin domains with PRC1 substructures inside the cell nucleus is specific to polycomb-associated chromatin, we performed similar experiments with oligopaint probes covering single or multiple domains of void chromatin (called black chromatin in Supplementary Fig. S7c, d), which is repressed but not associated with the polycomb machinery. In contrast to polycomb-associated chromatin, void chromatin was not associated with Ph nuclear substructures (Supplementary Fig. S7c, d).

### PRC1 nanoglobules may explain Ph-dependent BX-C compaction

**Fig. 6:**
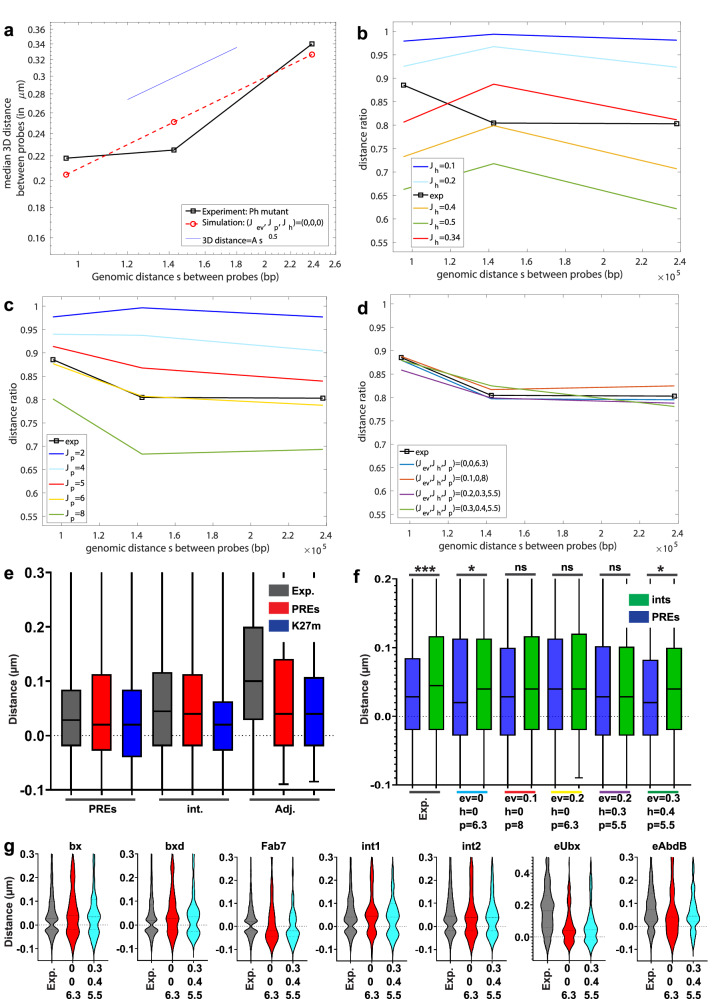
Interactions between PREs and PRC1 nanoglobules can mimic Ph-dependent BX-C compaction. **a** Median 3D distance between FISH probes as a function of the genomic distance between them, measured experimentally (black squares) or simulated using monomers with a 24 nm radius (red circles). The thin blue line represents the scaling law ${distance} \sim {s}^{1/2}$, which is expected for an equilibrated homopolymer model. **b**–**d** Ratio of the median distances between real or simulated wild-type (WT) and Ph-mutant conditions. Simulated WT conditions correspond to different sets of parameters (${J}_{{ev}}$, ${J}_{h}$, ${J}_{p}$), and the Ph mutant-like condition always corresponds to (${J}_{{ev}}$, ${J}_{h}$, ${J}_{p}$)=($0$, $0$, $0$), as in **a**. **b** We set ${J}_{{ev}}=0$ and ${J}_{p}=0$ and vary ${J}_{h}$. **c** We set ${J}_{{ev}}=0$ and ${J}_{h}=0$ and vary ${J}_{p}$. **d** As in **b**, **c**, but for different parameter sets with ${J}_{{ev}}\ne 0$ that fit the data well. **e** Box plots showing the distribution of minimum distances between the center of FISH spots and the border of Ph substructures measured in images from experiments (exp., in gray) or simulations using 2 parameter sets (${J}_{{ev}}$, ${J}_{h}$, ${J}_{p}$) = ($0$, $0$, $6.3$) for PREs (in red) and (${J}_{{ev}}$, ${J}_{h}$, ${J}_{p}$) = ($0$, $0.34$, $0$) for K27m (in blue). **f** Box plots comparing the positions of PREs and intermediate regions relative to the border of Ph substructures in the experimental data and 5 parameter sets computed with increasing exclusion volume. ns, not significant; *P < 0.05; ***P < 0.001. **g** Violin plots presenting data for each individual probe and focusing on the experimental results and the 2 parameter sets (${J}_{{ev}}$, ${J}_{h}$, ${J}_{p}$) = ($0$, $0$, $6.3$) and (${J}_{{ev}}$, ${J}_{h}$, ${J}_{p}$) = (0.3, 0.4, 5.5), which optimally reproduce significant differences between PREs and intermediate regions.

To test whether the association of PcG target loci with PRC1 nanoglobules might explain the previously reported Ph-mediated compaction of BX-C^21^, we used a polymer modeling approach. A 400 kb-long chromatin region encompassing the BX-C locus was modeled as a polymer in which individual monomer states were informed by published ChIP-seq profiles^37^. As monomers, we defined 1-kbp beads with one of three states, namely, a PRE, an H3K27me3, or a neutral state, depending on whether a PRE was located within the genomic coordinates assigned to the bead or whether the bead coordinates corresponded to a region covered by H3K27me3 (Supplementary Fig. S8a). The 3D distances between the promoters of Ubx, AbdA, and AbdB measured in Ph mutant embryos^21^ were used to simulate the 3D configurations of the BX-C locus in the absence of PRC1 nanoglobules and to define the physical size of the monomers (Fig. 6a). A representative subset of 15 large Ph foci characterized in Fig. 1 was used to reconstruct artificial PRC1 structures by modeling each nanoglobule as a 70 nm diameter Gaussian sphere (shown in Supplementary Fig. S8b). To calculate the interaction of chromatin with PRC1 nanoglobules, we used three parameters: the nanoglobule exclusion volume (*Jev*) and 2 interaction potentials: one between the nanoglobules and PRE monomers (*Jp*) and another between the nanoglobules and H3K27me3 monomers (*Jh*) (Supplementary Fig. S8c, d). To optimize these parameters, we fitted our model to the effect of Ph on the physical 3D distances measured between the promoters of Ubx, AbdA, and AbdB^21^. Interestingly, if chromatin is fully soluble in PRC1 nanoglobules (i.e., in the absence of an excluded volume between chromatin and PRC1 nanoglobules, *Jev* = 0), accounting for interactions between only H3K27me3 monomers and nanoglobules (*Jp* = 0) cannot capture the Ph-dependent compaction of BX-C (Fig. 6b). In contrast, applying interactions only between PRE monomers and nanoglobules (*Jh* = 0) was sufficient to mimic the effect of Ph on BX-C compaction (Fig. 6c). By adding steric hindrance to the nanoglobules (*Jev* > 0), best fits require both the interactions of the PREs and the interactions of H3K27me3 monomers with the nanoglobules, with a dominant role played by PRE interactions (Fig. 6d).

To further investigate our model, we computed virtual images of Ph nanoglobules coupled with FISH spots to produce simulated image counterparts of the immuno-FISH experiments described in Fig. 2 (examples shown in Supplementary Fig. S8e). As in the experiments, we used these in silico images to measure the minimum distances between the center of the FISH spots and the boundary of the Ph substructures and compared these in silico measures with the experimental data. The set of parameters displaying only interactions between H3K27me3 and nanoglobules matched less precisely with the experimental data, notably because of its inability to reproduce the differences between PREs and intermediate regions (Fig. 6e). Although several parameter sets were able to mimic the effect of Ph on BX-C compaction, only two of them were able to reproduce a significant difference between the localization of PREs and intermediate regions with respect to Ph substructures (Fig. 6f). The first relies solely on the interactions between PREs and nanoglobules, which are modeled in the absence of excluded volume (*Jev* = 0; *Jh* = 0 and *Jp* = 6.3 k_B_T). The second requires a weak interaction with H3K27me3 to balance steric hindrance, in addition to a strong interaction between the nanoglobules and PREs (*Jev* = 0.3 k_B_T; *Jh* = 0.4 k_B_T and *Jp* = 5.5 k_B_T) (Fig. 6f). We further extended this analysis to identify crucial parameters responsible for this compaction. Scatterplots of the minimum distances between the center of FISH spots and the boundary of the Ph substructures (*X* axis) with the mean distance between nanoglobules (i.e., the size of foci, on the *Y* axis) revealed no correlation (Supplementary Fig. S9a–b). In contrast, the density of nanoglobules within the large PRC1 foci (Supplementary Fig. S9c, d), as well as the intensity of Ph nanoglobules (Supplementary Fig. S9e, f), were correlated with the distance between FISH spots and the boundary of the Ph substructures. These observations suggest that the spatial density of Ph nanoglobules, as well as their PRC1 concentration, increases overall BX-C compaction.

An examination of each locus individually indicated that PREs (bx, bxd and Fab7) exhibit similar behavior, as do intermediate regions (int1 and int2) (Fig. 6g). Only the adjacent regions showed some discrepancies: although both sets of parameters matched relatively well with the experimental data for the region adjacent to *Abd-B* (eAbdB), they were unable to reproduce the positioning of the region adjacent to *Ubx* (eUbx) relative to Ph substructures (Fig. 6g). These observations suggest that chromatin insulators might play a role in preventing the association of adjacent chromatin with PRC1 nanoglobules and should not be modeled as neutral monomers. This possibility is consistent with the fact that H3K27me3 spreads toward eAbdB, which has weak but significant levels of H3K27me3 and is included in the topologically associating domain (TAD) that encompasses the BX-C locus, whereas H3K27me3 halts abruptly at the end of the *Ubx* gene, at a position that coincides with the proximal boundary of the BX-C TAD^38^ (see Supplementary Fig. S2a).

To evaluate the influence of PRC1 nanoglobule size on BX-C folding, we performed simulations with 280 nm nanoglobules — instead of the 70 nm that corresponds to the actual size of the nanoglobules observed by STED microscopy — and the parameters *Jev* = 0, *Jh* = 0, and *Jp* = 6.3 k_B_T, which were previously optimized. With 280 nm nanoglobules, the difference in location between PREs and intermediate regions relative to the border of the PRC1 substructures was no longer significant, and the positions of PREs and intermediate regions relative to the PRC1 substructures were strongly altered (Supplementary Fig. S9g). These data suggest that the organization in small nanoglobules is a strong determinant of the relative organization of PRC1 on its target chromatin. Finally, to test the effect of the interaction force between PREs and PRC1 nanoglobules, we used the Ph ChIP profile to modulate the intensity of the PRE interaction and the parameters *Jev* = 0.3 k_B_T, *Jh* = 0.4 k_B_T, and *Jp* = 5.5 k_B_T. Unlike in the initial simulation, in which all the PREs were identical, the difference in localization between PREs and intermediate regions relative to the PRC1 nanoglobules decreased as the intensity of the PRE interaction depended on the ChIP profile (Supplementary Fig. S9h). This finding indicates that, in our simulations, the linear localization of PREs is more important than the level of PRC1 bound to PREs. Taken together, these results indicate that chromatin interaction with PRC1 nanoglobules can recapitulate Ph-mediated BX-C compaction and clearly designate PREs as major elements driving polycomb-associated 3D chromatin architecture. The density of nanoglobules within the large PRC1 foci, the intensity of Ph labeling, and the size of Ph nanoglobules are key parameters that controlled BX-C folding in our simulations. Furthermore, polymer simulations provided evidence that PRC1 nanoglobules, the genomic position of PRC1 subunits, and the in situ localization of polycomb-associated chromatin relative to PRC1 nanoglobules cooperate to drive BX-C folding via the canonical polycomb machinery.

## Discussion

By imaging PRC1 subunits in *Drosophila* embryos by STED microscopy, we showed that large polycomb foci are composed of multiple substructures, which we named PRC1 nanoglobules. The use of single-cell imaging approaches, such as microscopy, allowed us to demonstrate that the association of repressed *Hox* chromatin with PRC1 nanoglobules is probabilistic. This is impossible to achieve with sequencing-based methods, such as ChIP, because they either involve bulk analysis of cell populations or, in the case of single cells, their low sensitivity does not allow one to conclude whether the absence of a signal means the absence of molecular association or the loss of molecules during the procedure. Furthermore, real-time imaging results showing rapid movement of PRC1 nanoglobules are consistent with the possibility that the observed probabilistic association of PRC1 with its target chromatin in FISH might be explained by the dynamic association of PRC1 with its targets in each cell.

Surprisingly, the localization of individual FISH probes, as well as the imaging of the entire BX-C locus, suggests that *Hox* chromatin often localizes at the periphery of PRC1 nanoglobules. This is unexpected, and given that the observations are based on imaging, which has limited spatial resolution, a legitimate question is whether alternative explanations exist that cannot be excluded because of limitations in the imaging approach. One question is whether chromatin might fit inside PRC1 nanoglobules in vivo. To answer this question, the number of PRC1 complexes and nucleosomes associated with a PRC1 nanoglobule can be estimated using previously published descriptions of the number of PRC1 complexes per embryonic nucleus and the number of nucleosomes per kb in *Drosophila*^39,40^. If one assumed that nucleosomes and PRC1 complexes could condense maximally, i.e., by filling the entirety of the volume of a nanoglobule and excluding everything else, their volume would be equivalent to a sphere with a diameter similar to the size of PRC1 nanoglobules measured by STED microscopy (Supplementary Fig. S10). However, this finding is in stark contrast with the estimated percentage of nuclear volume occupied by chromatin, which is less than 10%^41,42^, as well as with the observation that in vitro reconstituted PRC1-occupied chromatin is porous to molecules of a diameter up to 16 nm (~600 kDa) and that it does not perturb accessibility to proteins of > 100 kDa^22^. Together, these data strongly suggest that the volume of a PRC1 nanoglobule cannot be entirely occupied by the PRC1 complexes and nucleosomes. Instead, PRC1 and nucleosomes might occupy a minor proportion of the total available space, leading to the conclusion that most of the chromatin within polycomb domains must be located at the periphery of PRC1 nanoglobules, which is consistent with our observations at *Hox* loci.

While previous imaging of PRC1 subunits inside *Drosophila* cell nuclei using superresolution microscopy described the formation of nuclear structures with sizes below the limit of spatial resolution of confocal microscopy, they did not report the presence of foci composed of multiple PRC1 nanoglobules^43^. Here, we exploited the large genome size of *Hox* clusters^11,12^, enabling us to identify multiple substructures. Unlike *Hox* clusters and other large target loci that contain multiple target genes and are bound at multiple sites by polycomb complexes, most small H3K27me3 chromatin domains are likely associated with a single PRC1 nuclear structure, as most PRC1 nanoglobules are isolated in the cell nucleus. However, 30% of them form spatial groups, suggesting that the organization of clustered polycomb nanoglobules applies to a sizeable fraction of the polycomb target chromatin.

The observation of PRC1 nanoglobules is consistent with the ability of some PRC1 subunits to phase separate^24–26,28,44–46^. To minimize energy, it is thought that multiple condensates tend to gradually merge into larger condensates, but our observations showing that large polycomb foci are always made up of several PRC1 nanoglobules suggest that some mechanisms prevent this fusion. Live imaging shows rapid movement of Ph substructures. These fast motions might counteract liquid-liquid phase separation (LLPS) forces and prevent the fusion of nanoglobules into one large condensate. Accordingly, a recent study reported unfused PRC1‒chromatin condensates in vitro that did not ripen into large single droplets and that depended on the polymerization of a mammalian homolog of the Ph subunit of cPRC1^27^. Furthermore, solid particles inside Pickering emulsions or molecules binding to the surface of the condensates, acting as surfactants, might block the fusion of the latter^47,48^. Overall, the nonfusion of nanoglobules into large polycomb foci may be a signature of complex physicochemical mechanisms possibly counteracting LLPS-driven fusion. Alternatively, the formation of nanoglobules observed by STED microscopy might result from a more conventional model in which PRC1 subunits bind directly to PREs, promoting the establishment of loops between them^8,49–51^. However, our results do not favor this model because chromatin nanoglobules and PRC1 nanoglobules form different structures, which is consistent with a more dynamic mode of PRC1 binding to its target sites that involves regulated condensate formation.

Until now, it has been difficult to link the position of PRC1 subunits on chromatin and their localization in the cell nucleus because of the resolution gap between genomic and microscopic approaches. Imaging of immuno-FISH experiments by STED microscopy allowed us to fill this gap by revealing a difference between the localization of PREs and that of PRE-flanking chromatin coated with H3K27me3, which was never specifically compared with the localization of PRC1 subunits in imaging experiments^11,12^. On the basis of the ChIP profiles of PRC1 subunits and H3K27me3 and the position of PRC1 nanoglobules within large polycomb foci, polymer modeling could predict chromatin architectures produced by Ph-mediated BX-C compaction^21^. Previous studies investigating the effects of the polycomb machinery on chromatin compaction have reported conflicting findings^1–3^ and significant intercellular variability^21,23,41,52–55^. However, they focused only on chromatin, whereas the inclusion in this work of the analysis of PRC1 proteins and of the positioning of chromatin regions relative to PRC1 nanoglobules allowed us to address the effect of the canonical polycomb machinery on 3D folding of their target chromatin. Consistent with the key role of PREs^3,7^, polymer simulations indicate that they constitute the architectural elements leading to the interaction of polycomb target loci with PRC1 nanoglobules. However, PRC1 nanoglobules also contain H3K27me3-coated chromatin, underscoring the role of chromatin adjacent to PREs in polycomb function. The association of neighboring chromatin with PRC1 nanoglobules could help explain why PREs cannot be simply inferred from the genomic sequence^10,56^ and why deletions of all PREs at a locus do not always abolish polycomb recruitment^9^. Perhaps the cooperative association of cryptic polycomb binding sequences with PRC1 nanoglobules, together with their intervening regions, might simultaneously stabilize PRC1 binding and favor the coating of whole loci with H3K27me3. Taken together, these data identify PRC1 nanoglobules as key physical links between PcG protein binding to individual target sites and the formation of genomic domains that mediate stable gene silencing of large target loci such as *Hox* clusters.

## Materials and methods

### Fly lines and embryo fixation

The Oregon-R *w*^1118^ line was used as the WT control line. The *ph*^*del*^ stock is a null mutant^57^ and was balanced over the *KrGFP-FM7c* balancer (FKG: obtained from BL#5193 of the Bloomington *Drosophila* Stock Center). The *Pc*^*XT109*^ stock is a null mutant^58^ and was balanced over the *KrGFP-TM3, Sb* balancer (TKG: obtained from BL#5195 of the Bloomington *Drosophila* Stock Center). Pc immunolabeling allowed us to discriminate the mutants from the controls since approximately 25% of the embryos were devoid of Pc during the elongated stages of the germ band. The work with transgenic strains of *Drosophila* was performed under ethical approval no. 13462 of the Ministre de l’Enseignement Supérieur, de la Recherche et de l’Innovation, issued on 1 July 2025. The Ph-D_eGFP, Psc_eGFP, Pc_eGFP, and Sce_eGFP lines were obtained by CRISPR technology using a 1 guide strategy. Guides were designed before the stop codon. A GS linker and an eGFP tag were inserted at the C-terminus in fusion with the sequence coding for the proteins. The construction and injection were performed by Rainbow Transgenic Flies. The constructs were assembled in a pHD vector (addgene plasmid #51434). The pHD vector and vector-containing guide were injected into the embryos of the Cas9 fly line. Successful genomic integration events were visualized through the expression of the floxed dsRed cassette. The dsRed cassette of the Ph-D_eGFP line was further excised by Cre-mediated recombination between lox sites.

Guide list:

- Ph-D_eGFP: taatcctttacatcgcctgg cgg
- Psc_eGFP: cgg ccaccaaaagcaagtga
- Pc_eGFP: tgggtcgaagctcaagctac tgg
- Sce_eGFP: gtacgaagcgttttagtatt agg

To image Psc-GFP, Sce-GFP, and Pc-GFP in *ph*^*del*^ embryos, we crossed homozygous males carrying a GFP-PRC1 subunit with *ph*^*del*^/FKG females. As expected, a quarter of the embryos showed no nuclear foci, whereas the others showed an accumulation of Psc-GFP, Sce-GFP, or Pc-GFP within the cell nucleus. Flies were maintained on standard cornmeal yeast extract media at 21 °C. The embryos were subsequently harvested on agar/vinegar plates. The embryos were fixed according to the protocol described by Bantignies and Cavalli^59^. Briefly, embryos were dechorionated with bleach for 5 min and transferred into a glass flask, after which 5 mL of fixation buffer, containing 4% paraformaldehyde, KCl (60 mM), NaCl (15 mM), spermidine (0.5 mM), spermine (0.15 mM), EDTA (2 mM), EGTA (0.5 mM), and PIPES (15 mM), and 5 mL of heptane was added. The embryos were fixed under vigorous agitation on a mini-shaker for 25 min. The aqueous phase was then removed, and 5 mL of methanol was added. After shaking for 1 min, the fixed embryos were collected at the bottom of the glass flask and stored in methanol at −20 °C.

### Immuno-DNA FISH and immunolocalization

Immuno-DNA FISH experiments were performed according to the protocol described by Bantignies and Cavalli^59^. Briefly, to rehydrate fixed embryos, we used a series of methanol/PBS + 0.1% tween (PBT) solutions: 100/0; 90/10; 70/30; 50/50; 30/70; and 0/100. The embryos were then incubated in a solution of RNase A (200 µg/mL) in PBT for 2 h. After an additional 2 h and 30 min of incubation in PBS + 0.25% Triton X-100 (PBTr), the embryos were progressively transferred to a prehybridization mixture (pHM) containing 50% formamide, 4× SSC, 100 mM NaH_2_PO_4_ and 0.1% tween. The embryos were incubated for 20 min in a series of PBTr/pHM solutions: 80/20; 50/50; 20/80; and 0/100. A denaturation step was performed by incubating the embryos in pHM solution for 15 min at 80 °C. Then, hybridization was performed for 16–20 h at 37 °C in a solution containing deionized 50% formamide, 2× SSC, 10% dextran sulfate, 0.5 mg/mL salmon sperm DNA and labeled probes. The embryos were subsequently washed twice for 20 min at 37 °C in a solution containing 50% formamide, 2× SSC, and 0.3% CHAPS. Finally, the embryos were transferred to PBT solution by progressively decreasing the concentration of formamide.

The primers used to synthesize the DNA FISH probes are listed in Supplementary Table S1. Immuno-DNA FISH experiments were performed using either an anti-Pc rabbit polyclonal antibody (1/100 dilution) or an anti-Ph goat polyclonal antibody (1/250 dilution) developed in our laboratory^34^ as primary detection antibodies with StarRed-conjugated secondary antibodies (anti-rabbit: Abberior; STRED-1002-500UG; 1/100 dilution or anti-goat: Abberior; STRED-1055-500UG; 1/100 dilution). Single-locus FISH probes were directly labeled with Alexa 594 ( ~ 7 pmol/µL for a DNA concentration of ~85 ng/µL). Typically, we added 1.7 µL of probes to 40 µL of hybridization buffer. To detect the entire BX-C domain (Fig. 5), polycomb-associated domains or void chromatin domains (Supplementary Fig. S7), we used oligopaint probes coupled to Alexa 647 and performed the same FISH and immuno-FISH protocols. The BX-C oligopaint probe was made using the 89D–89E/BX-C library consisting of 42-mer sequences discovered by OligoArray 2.1^60^. The single domain associated with polycomb is located on chromosome 2 L (genomic coordinate: 11317986–11468388), the multiple domains associated with polycomb are located on chromosome 3 R (genomic coordinates: 2496062–2870013; 6342956–6500513; 8066650–8143013; 12199694–12252379), the single void chromatin domain is located on chromosome 2 L (genomic coordinate: 10540472–10717954), and the multiple void chromatin domains are located on chromosome 3 R (genomic coordinates: 2295136–2461552; 4204574–4332372; 6734612–6947878; 8556860–8764131; 9966695–10042715; 11523771–11601926)^36^. We used an anti-goat Alexa 594-conjugated secondary antibody (Thermo Fisher Scientific; A-11058; dilution 1/100) to reveal Ph immunolabeling in immuno-FISH experiments performed with oligopaint probes labeled with A647.

To perform immunolabeling experiments, we rehydrated fixed embryos using a series of methanol/PBS + 0.1% tween (PBT) solutions: 100/0; 90/10; 70/30; 50/50; 30/70; and 0/100. Then, the embryos were permeabilized by incubating them in PBS + 0.25 Triton X-100 (PBTr) for 2.5 h. A solution of PBS containing 2% BSA (A7906-100G; Sigma‒Aldrich) was used to block the nonspecific fixation of antibodies, and the embryos were incubated for 2 h in this solution. We used the same primary antibodies at the same concentrations as those used in the immuno-FISH experiments, and we diluted them in a PBTr solution to incubate the embryos overnight at 4 °C. After 5 washes in PBTr for 20 min, we used StarRed-conjugated secondary antibodies (anti-rabbit: Abberior; STRED-1002-500UG; dilution 1/100 or anti-goat: Abberior; STRED-1055-500UG; dilution 1/100) to detect either Ph or Pc immunolabeling. For the Pc and Ph double immunolabeling experiments, we used A594-conjugated anti-rabbit (Thermo Fisher Scientific; A-21207; dilution 1/100) and StarRed-conjugated anti-goat (Abberrior; STRED-1055-500UG; dilution 1/100) secondary antibodies. Secondary antibodies were diluted in PBTr and incubated for 2 h at room temperature. Finally, the embryos were washed 5 times in PBTr for 20 min. In both the immunolabeling and immuno-experiments, the embryos were mounted in Abberior liquid mounting medium (Abberrior; MM-2009-2X15ML) between slides and coverslips (18 × 18 mm; #1.5 glass Zeiss), and DAPI staining was not performed.

### Microscopy

AiryScan microscopy was performed using an LSM 980 microscope (Carl Zeiss Microscopy, Jena, Germany) with an AiryScan2 detector and a 63× PlanApo objective with a numerical aperture (N.A.) of 1.4. Ph-GFP was excited with a 488 nm laser diode, and we used the AiryScan SR mode to collect images with an optimal pixel size of 42 nm. The raw images were processed to produce AiryScan images with a Wiener filter automatically adjusted by the Zen software that controlled the microscope. For the timelapse experiments, we acquired 60 images of 376 × 226 pixels every 100 ms for 6 s.

STED images were acquired with an Abberior STED superresolution microscope (Abberior Instruments, Göttingen, Germany) equipped with a 100× Plan Superapochromat objective with an N.A. of 1.4. The Alexa 594 fluorochrome was excited with a 561 nm laser, whereas the StarRed or Alexa 647 fluorochromes were excited with a 640 nm laser. We collected images with a pixel size of 20 nm and used autofocus to correct the *Z* drift occurring during image acquisition. The pinhole was always set to 1 airy unit for maximal axial resolution. To compare STED and confocal microscopy, some images were acquired by switching between the STED and confocal mode for every line. For depletion, we used a 775 nm laser with a power of 3 watts. 2D images corresponded to a single plane, whereas 3D images consisted of 5 sections collected with a *Z*-step of 300 nm. Single Ph or Pc immunolabeling was detected with only the StarRed fluorochrome, and images were acquired using 30% laser power for depletion. Double immunolabeling was performed using 20% laser power for the depletion of StarRed to detect Ph and 25% laser power for the depletion of Alexa 594 to reveal Pc. We only collected 2D images of immuno-FISH experiments, and probes were always revealed with Alexa 594, while Ph/Pc immunolabeling was detected with the StarRed fluorochrome. We used 30% laser power to deplete both fluorochromes in Ph immuno-FISH experiments, whereas we used 20% depletion for StarRed and 30% depletion for Alexa 594 to image Pc immuno-FISH experiments. To image FISH experiments performed with Alexa 647-labeled oligopaint probes, we used a laser depletion of 4%. When FISH experiments performed with oligopaint probes were combined with Ph immunolabeling revealed with Alexa 594, we used a depletion of 4% laser power to deplete Alexa 647 and 12% for Alexa 594.

### Image analysis

To characterize Ph or Pc nanoglobules present in large Ph/Pc foci (Supplementary Fig. S1a), we used 3D images acquired by both confocal microscopy and STED microscopy. Despite the quality of the stage, mild drift might have occurred between the two sections. Because we used a pixel size of 20 nm, we cannot neglect this lateral drift. To correct this, we used Huygens Professional 23.10 software (Scientific Volume Imaging, NL) by applying the options Deconvolution/Object Stabilizer/Stabilize slices along Z/Align adjacent slices using cross correlation on only the STED channel with no interpolation, no rotation detection, no iterative filtering and a crop to the original size. Next, we used Imaris 9.8.0 software (Oxford Instruments, UK) for actual image quantification. The large Ph/Pc foci were segmented by applying the option surface with smooth 0.1 µm/background subtraction (image segmentation relying on local contrast) and a diameter of the largest sphere, which fit into the object of 0.250 µm on the confocal channel. To define these objects, we used a threshold with a background subtraction of 40 and the option split touching objects at 0.250 µm. Once identified, we kept objects with a quality above 15 and a number of voxels greater than 700. To identify Ph or Pc nanoglobules, we applied the “spots” option of Imaris software to the STED channel. We used the following parameters: an estimated diameter of 0.05 µm, an estimated *Z* diameter of 0.400 µm, a quality above 15, and background subtraction. Finally, using the option “spot within surface”, we assigned nanoglobules identified in STED images within large foci isolated in confocal images. The Imaris software can then easily compute the number of nanoglobules present in large foci, their intensity, and the maximum or minimum distances between them using the coordinates of the center of the spots. We used the Mann‒Whitney U test to compare distributions obtained under two different conditions.

To identify groups of Ph nanoglobules in 2D STED images, we used the “spots” option in Imaris software with the following parameters: an estimated diameter of 0.05 µm, a quality greater than 8.33, and background subtraction. Groups of nanoglobules were subsequently calculated using the “Split Spots” option, which groups all the spots within 140 nm of each other. To quantify the colocalization between Ph and Pc channels in confocal and STED images, we cropped 55 regions of interest (1 µm × 1 µm) containing one large focus. We used the option colocalization test in ImageJ to test whether the Ph and Pc signals were significantly colocalized (a *P* value above 0.95 indicated significant colocalization).

We only used 2D images acquired by STED microscopy to measure the distance of a single-FISH locus to the nearest Ph/Pc substructures. We applied a Gaussian filter with a radius of 1 pixel to the channel corresponding to the FISH signal, and we used the “spots” option of Imaris software to identify the centers of FISH loci. We applied the following parameters of the “spots” option: an estimated diameter of 0.04 µm with a quality above 3.11 for the Wi embryos and 1.5 for the control and PcXT109 embryos. To segment the Ph/Pc immunolabeling images, we used the “surface” option of Imaris software with the following parameters: no smooth background subtraction (local contrast) and a diameter of the largest sphere that fit into the objects of 0.1 µm. For the Wi embryos, we applied a threshold of 13 and a number of voxels greater than 30 for stages 9 to 13. Since many Ph/Pc substructures are small during stages 5 to 8, we set the number of voxels to greater than 5. As we performed double Ph and Pc immunolabeling to distinguish PcXT109 embryos from control embryos, Ph immunolabeling was slightly less intense than for Wi embryos. Therefore, we slightly decreased the surface segmentation parameter using a threshold of 11 and a number of voxels greater than 20 for the control and PcXT109 embryos. To measure the distance between the center of FISH loci and the border of Ph/Pc substructures, we applied the option “distance transformation” from surface objects (the first time by choosing “inside surface object” to calculate the distances inside Ph/Pc substructures, and the second time by choosing “outside surface object” to compute the distances of FISH loci located outside Ph/Pc substructures). To identify Ph/Pc substructures located less than 100 nm from FISH loci, we used the option “distance transformation” from the previously defined spots. Next, we selected “surface objects” located less than 100 nm from the FISH loci and extracted their surface area and intensity. To test whether FISH loci associated with small Ph/Pc substructures induce bias, we selected only surface objects with an area greater than 0.08 µm^2^ to remove small surfaces from the data collected inside the repressed regions of the Wi embryos from stages 9 to 13. Next, distances were identically measured, and we kept only distances < 0.5 µm. Considering all the Ph/Pc substructures or selecting only those with a surface greater than 0.08 µm^2^ produced similar results (data not shown). Similarly, using a threshold of 0.3 or 0.5 µm for the distance of a FISH locus to the nearest Ph/Pc substructure gave similar results (data not shown). The Violin plots in Figs. 2–3 were computed after the selection of surface objects greater than 0.08 µm^2^, and distances greater than 0.5 µm were removed. The plots in Fig. 4 and Supplementary Figs. S2, S3 and S6 show that all the surface objects computed and spaced greater than 0.3 µm have been removed. Finally, to compare the distributions of distances measured for each FISH probe, we used the Kruskal‒Wallis test (Figs. 2, 4c–h; Supplementary Figs. S2, S6e–h). In contrast, we used the Mann‒Whitney U test to compare distributions obtained under two different conditions (Figs. 3, 6; Supplementary Figs. S3, S6a–d and S9 g–h).

### Polymer simulations

We modeled the chromatin region (chr3R: 16,300,000–17,700,000) around the 7 probes used to study the Bithorax genomic region as a freely joined chain composed of *N* = 400 monomers, each of size *b* and encompassing 1 kbp of chromatin. Each bead *i* is associated with a PRE (${p}_{i}\in [0:1]$) and a H3K27me3 (${h}_{i}\in \{\mathrm{0,1}\}$) state if the corresponding region contains or is part of a PRE sequence or is enriched in H3K27me3 histone marks, respectively (Supplementary Fig. S8a). For a fixed set of PRC1 nanoglobules, we assume that each bead may interact with each nanoglobule through (1) steric hindrance (strength ${J}_{{ev}}$), (2) PRE-PRC1-mediated interactions (${J}_{p}$) and (3) H3K27me3-PRC1-mediated interactions (${J}_{h}$). The equilibrium structural properties of the chain are thus described by the Hamiltonian

$${H}_{{chain}}=\mathop{\sum }\limits_{k\in \{{NG}\}}\mathop{\sum }\limits_{i}{I}_{k}\left({J}_{{ev}}{f}_{{ev}}({r}_{i,k})-{(J}_{p}{p}_{i}+{J}_{h}{h}_{i}){f}_{\mathrm{int}}({r}_{i,k})\right)$$

where ${r}_{i,k}$ is the 3D distance between monomer *i* and the center of mass of nanoglobule, *k*, ${I}_{k}$ is the intensity of nanoglobule, *k*, and ${f}_{{ev}}(r)$ and ${f}_{\mathrm{int}}(r)$ are two decreasing functions modeling the interactions between a monomer and a nanoglobule (Supplementary Fig. S8c). To sample the equilibrium properties of such a Hamiltonian, we use the pruned-enriched Rosenbluth method (PERM)^61^. Briefly, the PERM is based on the Rosenbluth method, a biased-sampling algorithm in which a chain is iteratively grown monomer-by-monomer from monomer 1 to *N*, with the position of monomer *i* being biased toward the highest Boltzmann weights on the basis of the already placed monomers^62^. The pruning-enriching steps in the PERM allow enrichment of the sampling with configurations with high Boltzmann weights during chain growth, while pruning those with low weights^61^. Each configuration *c* generated by the PERM is associated with a Rosenbluth weight ${W}_{c}$ such that the thermodynamic average value of any variable $\Lambda (c)$ is given by $\left\langle \Lambda \right\rangle =[{\sum }_{c}\Lambda (c){W}_{c}]/[{\sum }_{c}{W}_{c}]$. In Supplementary Fig. S8d, we plotted configurations for an example with only one nanoglobule. For a more realistic PRC1 condensate distribution, for a fixed set of nanoglobules and a fixed set of parameters (${J}_{{ev}}$, ${J}_{h}$, ${J}_{p}$), we initiate the growth of 1000 configurations, randomly placing the first monomer in a sphere of radius 30*b* centered around the barycenter of the nanoglobule centers of mass to enforce the chain to remain near the nanoglobules. We repeat this sampling for ${N}_{g}$(15) sets of nanoglobules imaged by STED microscopy, for which we have extracted the mass positions of the nanoglobule centers (Supplementary Fig. S8b). For a given set of parameters, the overall average of any variable is given by the arithmetic mean over each set of nanoglobules.

We first considered the set (${J}_{{ev}}$, ${J}_{h}$, ${J}_{p}$) = ($0$, $0$, $0$), which corresponds to a situation without nanoglobules, i.e., a Ph mutation condition, to calibrate the model and infer the bead size *b*. From the set of grown configurations, we estimate the median 3D distances between the sets of monomers corresponding to the FISH probes used in the experiments. Since the PERM sample configurations have a normalized weight ${W}_{c}^{{\prime} }=\frac{{W}_{c}/{N}_{g}}{{\sum }_{c}{W}_{c}}$, the median of an arbitrary quantity $\Lambda ({\rm{c}})$ is defined by ranking all the obtained $\Lambda$ values in increasing order $\left\{\Lambda \left({c}_{0}\right)\le \Lambda \left({c}_{1}\right)\le \ldots \le \Lambda \left({c}_{T}\right)\right\}$ and taking the value $\Lambda \left({c}_{K}\right)$ such that ${\sum }_{i=1}^{K}{W}_{{c}_{i}}{\prime} \le 0.5\,\& {\sum }_{i=1}^{K+1}{W}_{{c}_{i}}{\prime} > 0.5$. As expected for a freely joined chain, we observed that the 3D distance was proportional to the square root of the genomic distance between FISH probes. By mapping our predictions to experiments (Fig. 6a), we found that $b=24{nm}$ best fit the experimental data by minimizing the sum of the squared differences between the predictions and experiments. Afterward, we fixed ${J}_{{ev}}=0$ and ${J}_{p}=0$ and asked whether interactions between the H3K27me3 region and nanoglobules alone could recapitulate the changes in relative distances between FISH probes between WT and Ph mutant conditions. We varied ${J}_{h}$ from 0 to $1$ K_B_T, computed the 3D median distances between probes in each case and the ratio of these median distances with the corresponding distances in Ph-mutant-like conditions ((${J}_{{ev}}$, ${J}_{h}$, ${J}_{p}$)=($0$, $0$, $0$)) and compared these ratios with the experimental estimation (Fig. 6b). Under these conditions, we could not find any ${J}_{h}$ value that could satisfy the experimental data. In contrast, by fixing (${J}_{{ev}}$, ${J}_{h}$) = ($0$, $0$), we found that having only interactions between PREs and nanoglobules (${J}_{p}\approx 6.3$ K_B_T) is sufficient to recapitulate the experimentally observed changes in distance (Fig. 6c). In the presence of weak excluded volume interactions (${J}_{{ev}}=0.1$ K_B_T), as previously described, only interactions with PRE (${J}_{p}\approx 8$ K_B_T were sufficient to capture experimental data, while interactions with the H3K27me3 region could not (data not shown). As we increased the excluded volume contribution (${J}_{{ev}}=0.2$ K_B_T or $0.3$ K_B_T), interactions with PRE alone could not explain the data but could be combined with interactions with the H3K27me3 region ([${J}_{p}\approx 5.5$ K_B_T, *J*_*h*_ ≈ 0.3 K_B_T] or [${J}_{p}\approx 5.5$ K_B_T, *J*_*h*_ ≈ 0.3 K_B_T], respectively) (Fig. 6d). Beyond ${J}_{{ev}}\ge 0.4$, no combination of ${J}_{p}$ and ${J}_{h}$ was able to capture the changes in median distances as quantitatively as in the cases of lower excluded volume interactions (data not shown).

We then asked if the different parameter sets that capture the observed changes in median distances between FISH probes ((${J}_{{ev}}$, ${J}_{h}$, ${J}_{p}$) = ($0$, $0$, $6.3$); ($0.1$, $0$, $8$); (0.2, 0, 6.3); ($0.2$, $0.3$, $5.5$); ($0.3$, $0.4$, $5.5$)) were compatible with the relative localization around the polycomb bodies of the seven BX-C sites studied experimentally in Fig. 2 and Supplementary Fig. S2. To do so, we first randomly chose snapshots generated for these parameters (10 per nanoglobule set) with a likelihood to be picked proportional to their normalized Rosenbluth weight. We then generated 2D images of each probe at 70 nm (or 280 nm for Supplementary Fig. S9g) spatial resolution and 20 nm pixel size by convoluting the positions in the (*X*-*Y*) plane of the monomers corresponding to the probe with a 2D Gaussian of standard deviation of 35 nm (or 140 nm for Supplementary Fig. S9g). To mimic the experimental conditions, we further blurred the channel corresponding to the nanoglobule images by applying a Gaussian filter with a radius of 0.04 µm. Afterward, we used the same procedure as that used with the experimental images to measure the distance of a FISH locus to the nearest Ph/Pc substructures.

## Supplementary information

Supplemental information

## Data Availability

The raw data used in this work are publicly available on the Zenodo repository under 10.5281/zenodo.16676358. 10.5281/zenodo.18669204

## References

[1] Tsompana, M. & Buck, M. J. Chromatin accessibility: a window into the genome. *Epigenetics Chromatin* **7**, 33 (2014). 25473421 10.1186/1756-8935-7-33 PMC4253006

[2] Panigrahi, A. & O’Malley, B. W. Mechanisms of enhancer action: the known and the unknown. *Genome Biol* **22**, 108 (2021). 33858480 10.1186/s13059-021-02322-1 PMC8051032

[3] Schuettengruber, B., Bourbon, H.-M., Di Croce, L. & Cavalli, G. Genome regulation by polycomb and trithorax: 70 years and counting. *Cell* **171**, 34–57 (2017). 28938122 10.1016/j.cell.2017.08.002

[4] Simon, J. A. & Kingston, R. E. Occupying chromatin: polycomb mechanisms for getting to genomic targets, stopping transcriptional traffic, and staying put. *Mol. Cell* **49**, 808–824 (2013). 23473600 10.1016/j.molcel.2013.02.013 PMC3628831

[5] Fischle, W. et al. Molecular basis for the discrimination of repressive methyl-lysine marks in histone H3 by Polycomb and HP1 chromodomains. *Genes Dev* **17**, 1870–1881 (2003). 12897054 10.1101/gad.1110503 PMC196235

[6] Blackledge, N. P. & Klose, R. J. The molecular principles of gene regulation by Polycomb repressive complexes. *Nat. Rev. Mol. Cell Biol.* **22**, 815–833 (2021). 34400841 10.1038/s41580-021-00398-y PMC7612013

[7] Kassis, J. A. & Brown, J. L. Polycomb group response elements in *Drosophila* and vertebrates. *Adv. Genet.* **81**, 83–118 (2013). 23419717 10.1016/B978-0-12-407677-8.00003-8 PMC4157523

[8] Ogiyama, Y., Schuettengruber, B., Papadopoulos, G. L., Chang, J.-M. & Cavalli, G. Polycomb-dependent chromatin looping contributes to gene silencing during *Drosophila* development. *Mol. Cell* **71**, 73–88.e5 (2018). 30008320 10.1016/j.molcel.2018.05.032

[9] De, S., Cheng, Y., Sun, M., Gehred, N. D. & Kassis, J. A. Structure and function of an ectopic Polycomb chromatin domain. *Sci. Adv.* **5**, eaau9739 (2019). 30662949 10.1126/sciadv.aau9739 PMC6326746

[10] Kahn, T. G. et al. Interdependence of PRC1 and PRC2 for recruitment to Polycomb Response Elements. *Nucleic Acids Res* **44**, 10131–10149 (2016). 10.1093/nar/gkw701 PMC5137424 27557709

[11] Schuettengruber, B. et al. Functional anatomy of polycomb and trithorax chromatin landscapes in *Drosophila* embryos. *PLoS Biol* **7**, e1000013 (2009). 19143474 10.1371/journal.pbio.1000013 PMC2621266

[12] Schwartz, Y. B. et al. Genome-wide analysis of Polycomb targets in *Drosophila* melanogaster. *Nat. Genet.* **38**, 700–705 (2006). 16732288 10.1038/ng1817

[13] Pauler, F. M. et al. H3K27me3 forms BLOCs over silent genes and intergenic regions and specifies a histone banding pattern on a mouse autosomal chromosome. *Genome Res.* **19**, 221–233 (2009). 19047520 10.1101/gr.080861.108 PMC2652204

[14] Buchenau, P., Hodgson, J., Strutt, H. & Arndt-Jovin, D. J. The distribution of polycomb-group proteins during cell division and development in *Drosophila* embryos: impact on models for silencing. *J. Cell Biol.* **141**, 469–481 (1998). 9548724 10.1083/jcb.141.2.469 PMC2148446

[15] Cheutin, T. & Cavalli, G. Progressive polycomb assembly on H3K27me3 compartments generates polycomb bodies with developmentally regulated motion. *PLoS Genet* **8**, e1002465 (2012). 22275876 10.1371/journal.pgen.1002465 PMC3262012

[16] Saurin, A. J. et al. The human polycomb group complex associates with pericentromeric heterochromatin to form a novel nuclear domain. *J. Cell Biol.* **142**, 887–898 (1998). 9722603 10.1083/jcb.142.4.887 PMC2132874

[17] Bantignies, F. et al. Polycomb-dependent regulatory contacts between distant Hox loci in. *Drosophila. Cell* **144**, 214–226 (2011). 21241892 10.1016/j.cell.2010.12.026

[18] Kundu, S. et al. Polycomb repressive complex 1 generates discrete compacted domains that change during differentiation. *Mol. Cell* **65**, 432–446.e5 (2017). 28157505 10.1016/j.molcel.2017.01.009 PMC5421375

[19] Gurgo, J. et al. Multiplexed chromatin imaging reveals predominantly pairwise long-range coordination between *Drosophila* Polycomb genes. *Cell Rep.* **43**, 114167 (2024). 38691452 10.1016/j.celrep.2024.114167

[20] Schoenfelder, S. et al. Polycomb repressive complex PRC1 spatially constrains the mouse embryonic stem cell genome. *Nat. Genet.* **47**, 1179–1186 (2015). 26323060 10.1038/ng.3393 PMC4847639

[21] Cheutin, T. & Cavalli, G. Loss of PRC1 induces higher-order opening of Hox loci independently of transcription during *Drosophila* embryogenesis. *Nat. Commun.* **9**, 3898 (2018). 30254245 10.1038/s41467-018-05945-4 PMC6156336

[22] Uckelmann, M. & Davidovich, C. Chromatin compaction by Polycomb group proteins revisited. *Curr. Opin. Struct. Biol.* **86**, 102806 (2024). 38537534 10.1016/j.sbi.2024.102806

[23] Murphy, S. E. & Boettiger, A. N. Polycomb repression of Hox genes involves spatial feedback but not domain compaction or phase transition. *Nat. Genet.***56**, 493–504 (2024).38361032 10.1038/s41588-024-01661-6

[24] Plys, A. J. et al. Phase separation of Polycomb-repressive complex 1 is governed by a charged disordered region of CBX2. *Genes Dev* **33**, 799–813 (2019). 31171700 10.1101/gad.326488.119 PMC6601514

[25] Tatavosian, R. et al. Nuclear condensates of the Polycomb protein chromobox 2 (CBX2) assemble through phase separation. *J. Biol. Chem.* **294**, 1451–1463 (2019). 30514760 10.1074/jbc.RA118.006620 PMC6364756

[26] Seif, E. et al. Phase separation by the polyhomeotic sterile alpha motif compartmentalizes Polycomb Group proteins and enhances their activity. *Nat. Commun.* **11**, 5609 (2020). 33154383 10.1038/s41467-020-19435-z PMC7644731

[27] Niekamp, S., Marr, S. K., Oei, T. A., Subramanian, R. & Kingston, R. E. Modularity of PRC1 composition and chromatin interaction define condensate properties. *Mol. Cell* **84**, 1651–1666.e12 (2024). 38521066 10.1016/j.molcel.2024.03.001 PMC11234260

[28] Kapur, I., Boulier, E. L. & Francis, N. J. Regulation of polyhomeotic condensates by intrinsically disordered sequences that affect chromatin binding. *Epigenomes* **6**, 40 (2022). 36412795 10.3390/epigenomes6040040 PMC9680516

[29] Williamson, I., Boyle, S., Grimes, G. R., Friman, E. T. & Bickmore, W. A. Dispersal of PRC1 condensates disrupts polycomb chromatin domains and loops. *Life Sci. Alliance* **6**, e202302101 (2023). 37487640 10.26508/lsa.202302101 PMC10366532

[30] Hug, C. B., Grimaldi, A. G., Kruse, K. & Vaquerizas, J. M. Chromatin architecture emerges during zygotic genome activation independent of transcription. *Cell* **169**, 216–228.e19 (2017). 28388407 10.1016/j.cell.2017.03.024

[31] Maeda, R. K. & Karch, F. The open for business model of the bithorax complex in. *Drosophila. Chromosoma* **124**, 293–307 (2015). 26067031 10.1007/s00412-015-0522-0 PMC4548009

[32] Bowman, S. K. et al. H3K27 modifications define segmental regulatory domains in the *Drosophila* bithorax complex. *Elife* **3**, e02833 (2014). 25082344 10.7554/eLife.02833 PMC4139060

[33] Pengelly, A. R., Kalb, R., Finkl, K. & Müller, J. Transcriptional repression by PRC1 in the absence of H2A monoubiquitylation. *Genes Dev* **29**, 1487–1492 (2015). 26178786 10.1101/gad.265439.115 PMC4526733

[34] Grimaud, C. et al. RNAi components are required for nuclear clustering of polycomb group response elements. *Cell* **124**, 957–971 (2006). 16530043 10.1016/j.cell.2006.01.036

[35] Cavalli, G. & Paro, R. The *Drosophila* Fab-7 chromosomal element conveys epigenetic inheritance during mitosis and meiosis. *Cell* **93**, 505–518 (1998). 9604927 10.1016/s0092-8674(00)81181-2

[36] Szabo, Q. et al. TADs are 3D structural units of higher-order chromosome organization in. *Drosophila. Sci. Adv.* **4**, eaar8082 (2018). 29503869 10.1126/sciadv.aar8082 PMC5829972

[37] Loubiere, V., Papadopoulos, G. L., Szabo, Q., Martinez, A.-M. & Cavalli, G. Widespread activation of developmental gene expression characterized by PRC1-dependent chromatin looping. *Sci. Adv.* **6**, eaax4001 (2020). 31950077 10.1126/sciadv.aax4001 PMC6954061

[38] Sexton, T. et al. Three-dimensional folding and functional organization principles of the *Drosophila* genome. *Cell* **148**, 458–472 (2012). 22265598 10.1016/j.cell.2012.01.010

[39] Bonnet, J. et al. Quantification of proteins and histone marks in *Drosophila* embryos reveals stoichiometric relationships impacting chromatin regulation. *Dev. Cell* **51**, 632–644.e6 (2019). 31630981 10.1016/j.devcel.2019.09.011

[40] Baldi, S. et al. Genome-wide rules of nucleosome phasing in. *Drosophila. Mol. Cell* **72**, 661–672.e4 (2018). 30392927 10.1016/j.molcel.2018.09.032

[41] Boettiger, A. N. et al. Super-resolution imaging reveals distinct chromatin folding for different epigenetic states. *Nature* **529**, 418–422 (2016). 26760202 10.1038/nature16496 PMC4905822

[42] Gelléri, M. et al. True-to-scale DNA-density maps correlate with major accessibility differences between active and inactive chromatin. *Cell Rep* **42**, 112567 (2023). 37243597 10.1016/j.celrep.2023.112567

[43] Wani, A. H. et al. Chromatin topology is coupled to Polycomb group protein subnuclear organization. *Nat. Commun.* **7**, 10291 (2016). 26759081 10.1038/ncomms10291 PMC4735512

[44] Akilli, N., Cheutin, T. & Cavalli, G. Phase separation and inheritance of repressive chromatin domains. *Curr. Opin. Genet. Dev.* **86**, 102201 (2024). 38701672 10.1016/j.gde.2024.102201

[45] Brown, K. et al. Principles of assembly and regulation of condensates of Polycomb repressive complex 1 through phase separation. *Cell Rep* **42**, 113136 (2023). 37756159 10.1016/j.celrep.2023.113136 PMC10862386

[46] Gemeinhardt, T. M. et al. A disordered linker in the Polycomb protein Polyhomeotic tunes phase separation and oligomerization. *Mol. Cell* **85**, 2128–2146.e15 (2025). 40441156 10.1016/j.molcel.2025.05.008 PMC12145237

[47] Kelley, F. M., Favetta, B., Regy, R. M., Mittal, J. & Schuster, B. S. Amphiphilic proteins coassemble into multiphasic condensates and act as biomolecular surfactants. *Proc. Natl. Acad. Sci. USA* **118**, e2109967118 (2021). 34916288 10.1073/pnas.2109967118 PMC8713756

[48] Folkmann, A. W., Putnam, A., Lee, C. F. & Seydoux, G. Regulation of biomolecular condensates by interfacial protein clusters. *Science* **373**, 1218–1224 (2021). 34516789 10.1126/science.abg7071 PMC8627561

[49] Eagen, K. P., Aiden, E. L. & Kornberg, R. D. Polycomb-mediated chromatin loops revealed by a subkilobase-resolution chromatin interaction map. *Proc. Natl. Acad. Sci. USA* **114**, 8764–8769 (2017). 28765367 10.1073/pnas.1701291114 PMC5565414

[50] Denaud, S. et al. A PRE loop at the dac locus acts as a topological chromatin structure that restricts and specifies enhancer–promoter communication. *Nat. Struct. Mol. Biol.* **31**, 1942–1954 (2024). 39152239 10.1038/s41594-024-01375-7 PMC11638067

[51] Cheutin, T. & Cavalli, G. The multiscale effects of polycomb mechanisms on 3D chromatin folding. *Crit. Rev. Biochem. Mol. Biol.* **54**, 399–417 (2019). 31698957 10.1080/10409238.2019.1679082

[52] Eskeland, R. et al. Ring1B compacts chromatin structure and represses gene expression independent of histone ubiquitination. *Mol. Cell* **38**, 452–464 (2010). 20471950 10.1016/j.molcel.2010.02.032 PMC3132514

[53] Grau, D. J. et al. Compaction of chromatin by diverse Polycomb group proteins requires localized regions of high charge. *Genes Dev* **25**, 2210–2221 (2011). 22012622 10.1101/gad.17288211 PMC3205590

[54] Boyle, S. et al. A central role for canonical PRC1 in shaping the 3D nuclear landscape. *Genes Dev* **34**, 931–949 (2020). 32439634 10.1101/gad.336487.120 PMC7328521

[55] Uckelmann, M. et al. Dynamic PRC1–CBX8 stabilizes a porous structure of chromatin condensates. *Nat. Struct. Mol. Biol.* **32**, 520–530 (2025). 39815045 10.1038/s41594-024-01457-6 PMC11919719

[56] Dorafshan, E., Kahn, T. G. & Schwartz, Y. B. Hierarchical recruitment of Polycomb complexes revisited. *Nucleus* **8**, 496–505 (2017). 28910569 10.1080/19491034.2017.1363136 PMC5703234

[57] Feng, S., Huang, J. & Wang, J. Loss of the Polycomb group gene *polyhomeotic* induces non-autonomous cell overproliferation. *EMBO Rep* **12**, 157–163 (2011). 21164514 10.1038/embor.2010.188 PMC3049426

[58] Franke, A., Messmer, S. & Paro, R. Mapping functional domains of the Polycomb protein of *Drosophila* melanogaster. *Chromosome Res.* **3**, 351–360 (1995). 7551550 10.1007/BF00710016

[59] Bantignies, F. & Cavalli, G. Topological organization of drosophila Hox genes using DNA fluorescent in situ hybridization. *Methods Mol Biol.* **1196**, 103–120 (2014). 25151160 10.1007/978-1-4939-1242-1_7

[60] Beliveau, B. J. et al. Single-molecule super-resolution imaging of chromosomes and in situ haplotype visualization using Oligopaint FISH probes. *Nat. Commun.* **6**, 7147 (2015). 25962338 10.1038/ncomms8147 PMC4430122

[61] Grassberger, P. Pruned-enriched Rosenbluth method: simulations of θ polymers of chain length up to 1 000 000. *Phys. Rev. E* **56**, 3682–3693 (1997).

[62] Rosenbluth, M. N. & Rosenbluth, A. W. Monte Carlo calculation of the average extension of molecular chains. *J. Chem. Phys.* **23**, 356–359 (1955).

