# Supplementary material for: PRC1 nanoglobules organize Hox chromatin during Drosophila embryogenesis
Source: Cell Discov. 2026 Jul 7;12:50. doi: 10.1038/s41421-026-00902-8 (PMC13338358; doi:10.1038/s41421-026-00902-8)
Supplement: Supplementary file 1 — Supplemental information [file 41421_2026_902_MOESM1_ESM.pdf]

Supplementary Information.

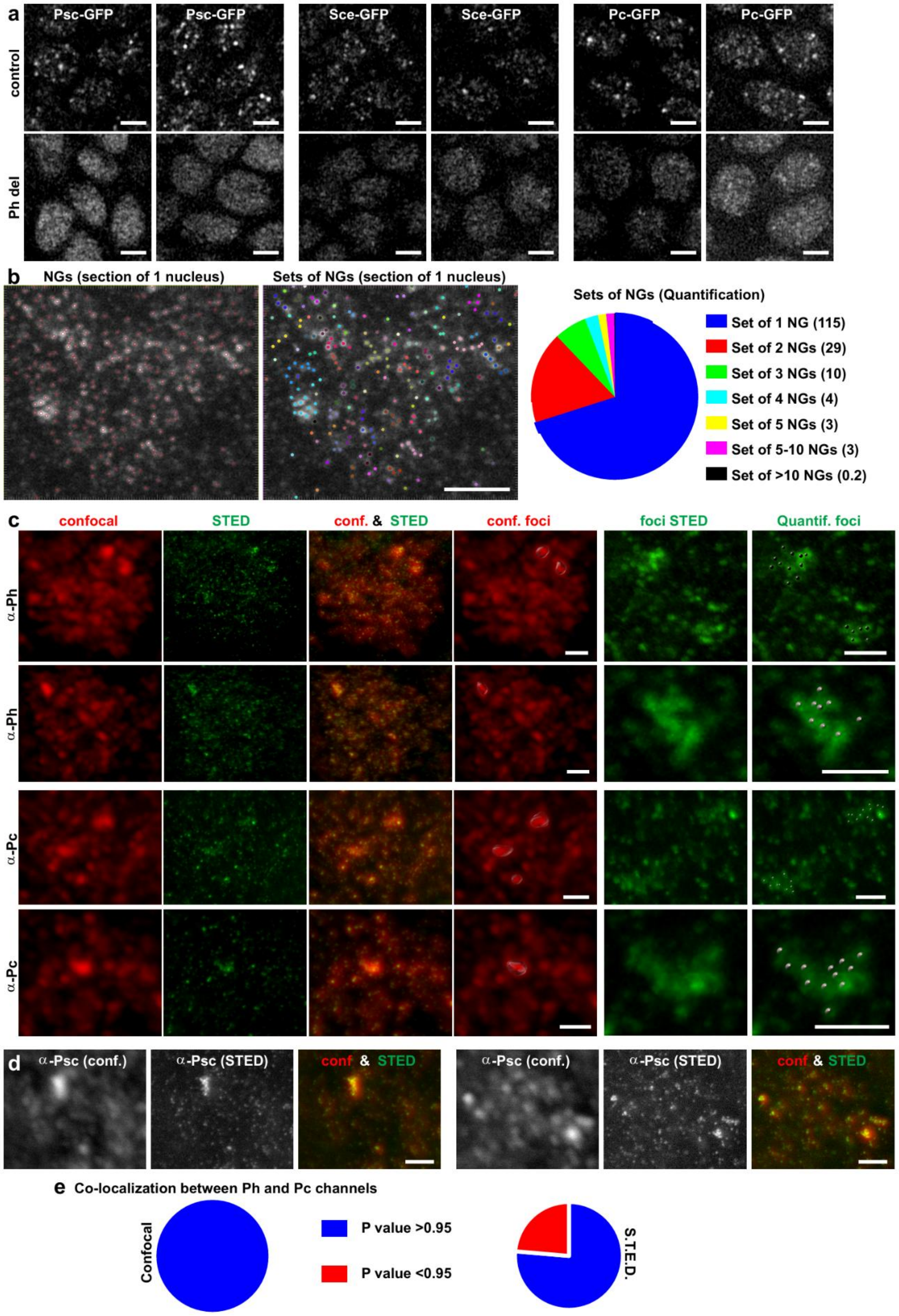

Supplementary Figure 1: Characterization of large PRC1 foci.

a: 2D pictures of Psc-GFP, Sce-GFP and Pc-GFP imaged in control and Ph del mutant embryos by AiryScan microscopy. While Psc-GFP, Sce-GFP and Pc-GFP form nuclear foci in control embryos, their nuclear distribution appears predominantly diffuse in embryos lacking the Ph subunit of PRC1.

Bars measure 2µm. **b**: Distribution of Ph nanoglobules inside the cell nucleus. Ph nanoglobules present in 2D images acquired by STED microscopy were segmented (an example is shown in the image on the left, the red circles indicating the segmented nanoglobules), and nanoglobules less than 140 nm apart were grouped together (the nanoglobules belonging to the same group are represented by disks of the same colour in the middle image). Although most of the nanoglobules are isolated (~70%), the remainder form groups containing several nanoglobules (pie chart on the right; N= 2786 nanoglobules in 16 nuclei). Bar measures 1µm. **c:** 3D projections of Ph or Pc immuno-labellings imaged in confocal (red) and STED (green) microscopies. Many faint Polycomb foci are difficult to isolate in confocal images, leading to rather diffuse nucleoplasmic signal, while small Polycomb foci appears as single structures well isolated from the nucleoplasm in STED images. In contrast, large foci can easily be segmented in confocal images (highlighted red objects in conf. foci). Bars measure 1 µm. The two rightmost columns (foci STED and Quantif. foci) show zoom-in images of large Polycomb foci. Bars measure 0.5 µm. Detection of local maxima identified substructures in STED images. To quantify nanoglobules (white dots) localized within large foci, we computed intersections between local maxima seen in STED and the large foci segmented in confocal images. **d:** Two examples of confocal, STED, and merged pictures of a Psc immuno-labelling acquired in the head of *Drosophila* embryos. Large Psc foci are also composed of several nanoglobules. Bars measure 1µm. **e:** Pie charts showing the quantification of Ph and Pc co-localization in confocal and STED images. Ph and Pc channels in confocal images are significantly co-localized in each large focus (P value >0.95), while Ph/Pc co-localization in STED images is not significant for a few large foci (P value <0.95). However, antibody accessibility and/or antigen masking may confound high-resolution co-localization and explain why Ph and Pc co-localization appears non-significant in some nanoglobules in STED microscopy.

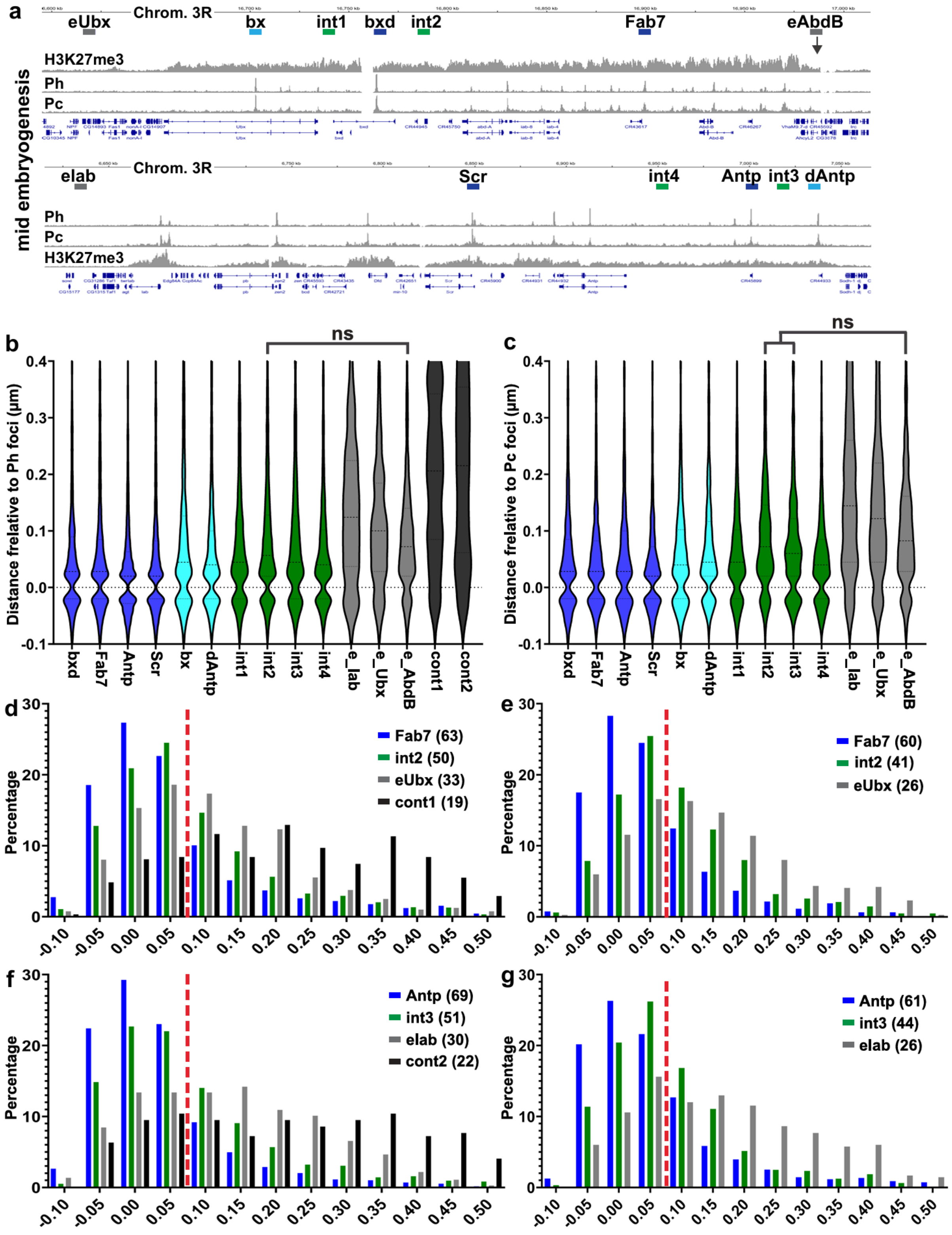

Supplementary Figure 2: Localization of Hox chromatin relative to Ph/Pc substructures.

a: Genomic maps showing the profiles of H3k27me3, Ph and Pc observed during mid-embryogenesis, and the localization of FISH probes in BX-C and ANT-C[1]. FISH probes detecting PREs are shown in blue, intermediate regions in green, and adjacent regions in grey. The arrow indicates the slight spreading of H3K27me3 towards the eAbdB probe. **b-c:** Violin plots showing the distribution of minimum distances measured between the center of FISH spots and the border of Ph (**b**) or Pc (**c**) substructures. These violin plots are missing data between +/- 20 nm because the minimum distance that can be measured is the size of a pixel (20 nm). Regions adjacent to Hox clusters (light grey) are located

further from Ph or Pc substructures than PREs positioned in the middle (dark blue) or periphery (light blue) of Hox clusters (p-values <0.0001). Regions adjacent to Hox clusters are also significantly ($p<0.0001$) less associated with Ph/Pc substructures than intermediates regions (green), except for e-AbdB which shows no significant difference compared to int2 with Ph/Pc substructures and int3 with Pc foci (ns). Repressed void chromatin (dark grey) rarely locates close to Ph substructures. **d-g:** Histograms of minimum distance measured between the center of FISH spots and the border of Ph (**d, f**) or Pc (**e, g**) substructures. These histograms compare probes located in/or adjacent to BX-C (**d-e**) or ANT-C (**f-g**). Fish spots are about 100 nm in diameter, so the center of those locate less than 50 nm away from immunolabelled substructures can touch them. Red dash-lines indicates the limit between FISH spots located in Ph/Pc substructures and the ones not associated with them. The percentage of FISH spots found associated with Ph/Pc staining are indicated in parentheses for each probe.

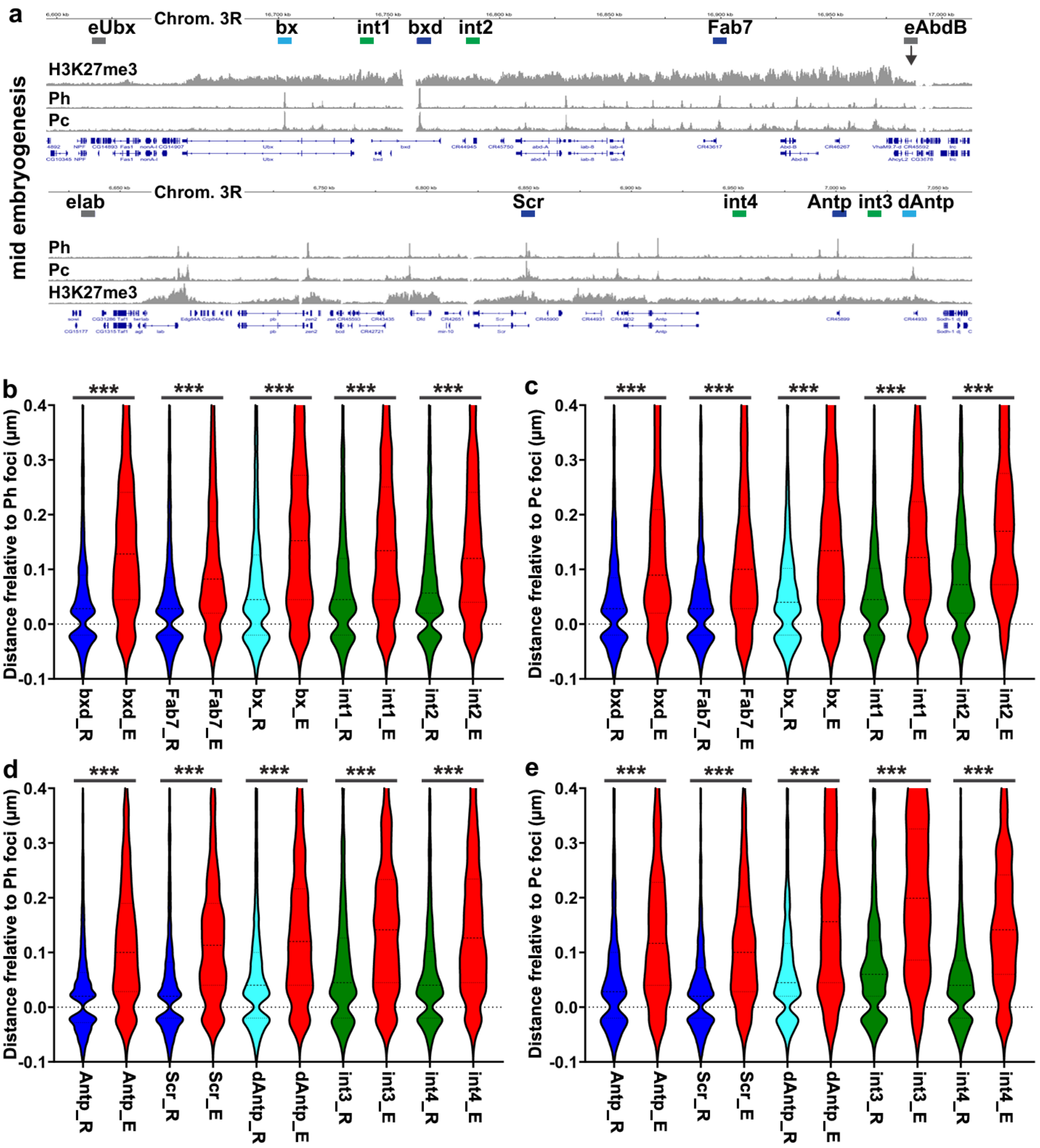

Supplementary Figure 3: Effect of transcription on the localisation of PREs and intermediate regions relative to Ph/Pc substructures.

a: Genomic maps showing the profiles of H3k27me3, Ph and Pc observed during mid-embryogenesis, and the localization of FISH probes in BX-C and ANT-C[1]. FISH probes detecting PREs are shown in blue, intermediate regions in green, and adjacent regions in grey. **b-e:** Effect of transcription on the localization of FISH probes located in BX-C (**b-c**) or ANT-C (**d-e**) relative to Ph (**b, d**) or Pc (**c, e**) substructures. In parasegments (PS) where the corresponding Hox gene is expressed (_E in caption: PS11-13 for int1, int2, bx and bxd; PS11-PS14 for Fab7; PS2 for Scr and PS4-5 for int3, int4, Antp and dAntp, see scheme in **Fig. 2b**), FISH spots of both PREs and intermediate regions are less associated with Ph/Pc substructures than in the head of embryos where each Hox gene is repressed (_R in caption). ***: $p<0.0001$.

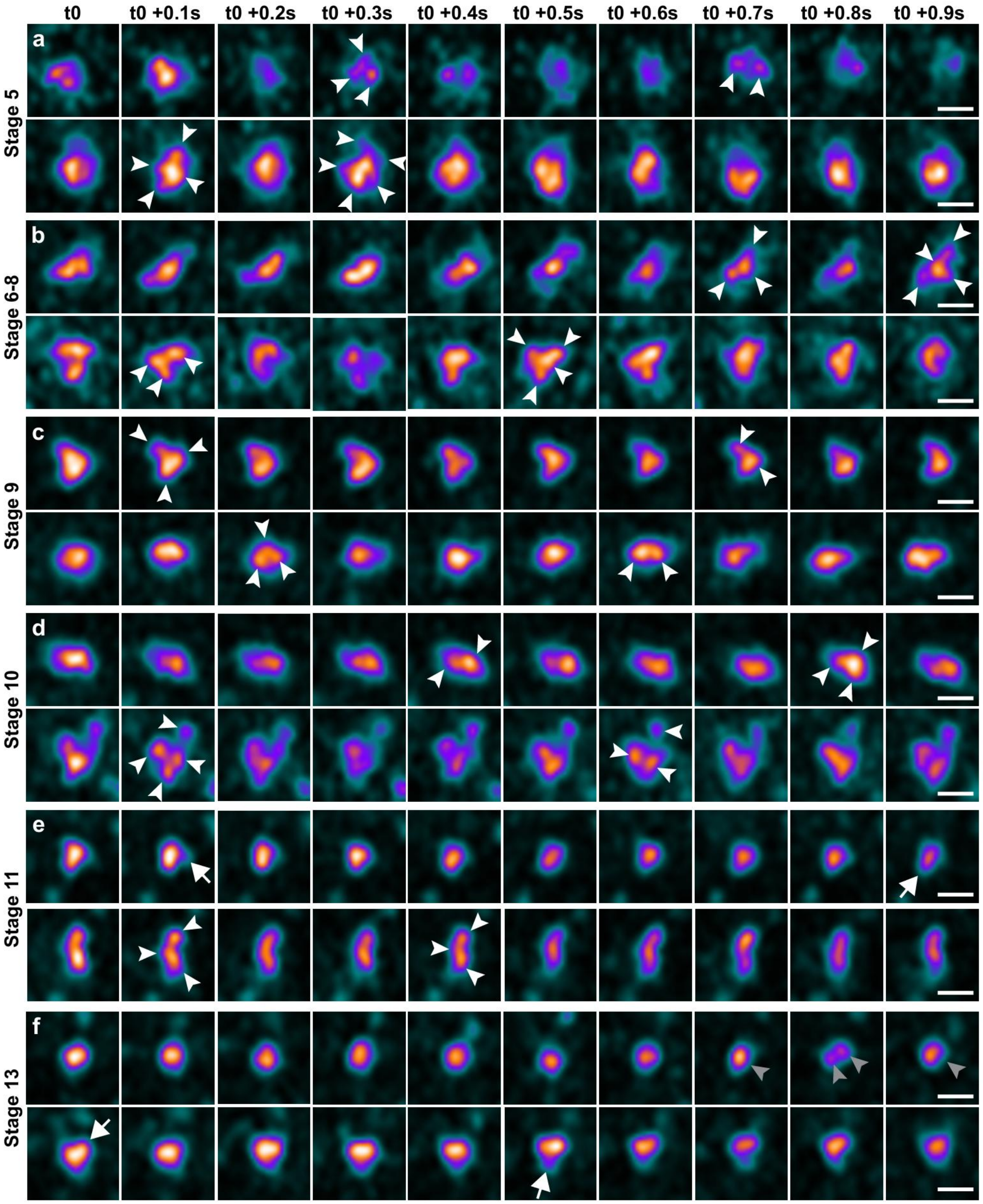

Supplementary Figure 4: Live imaging shows rapid movement of Ph substructures at several embryonic stages.

Pseudo-colours images of time-lapse experiments acquired by AiryScan microscopy visualizing large Ph foci located in the head of embryos expressing Ph-GFP at embryonic stages 5 (**a**), 6-8 (**b**), 9 (**c**), 10 (**d**), 11 (**e**) and 13 (**f**). Several mobile substructures can easily be observed during early embryogenesis (white arrowheads), whereas they become more difficult to discern during late embryogenesis (stages 11 and 13). Despite being smaller, the change in shape of Ph foci at stages 11 and 13 can still be appreciated in the movies. White arrows point few examples where Ph foci change shape, whereas grey arrowheads (in **f**) indicate a Ph focus dividing in two substructures. Bars measure 500 nm.

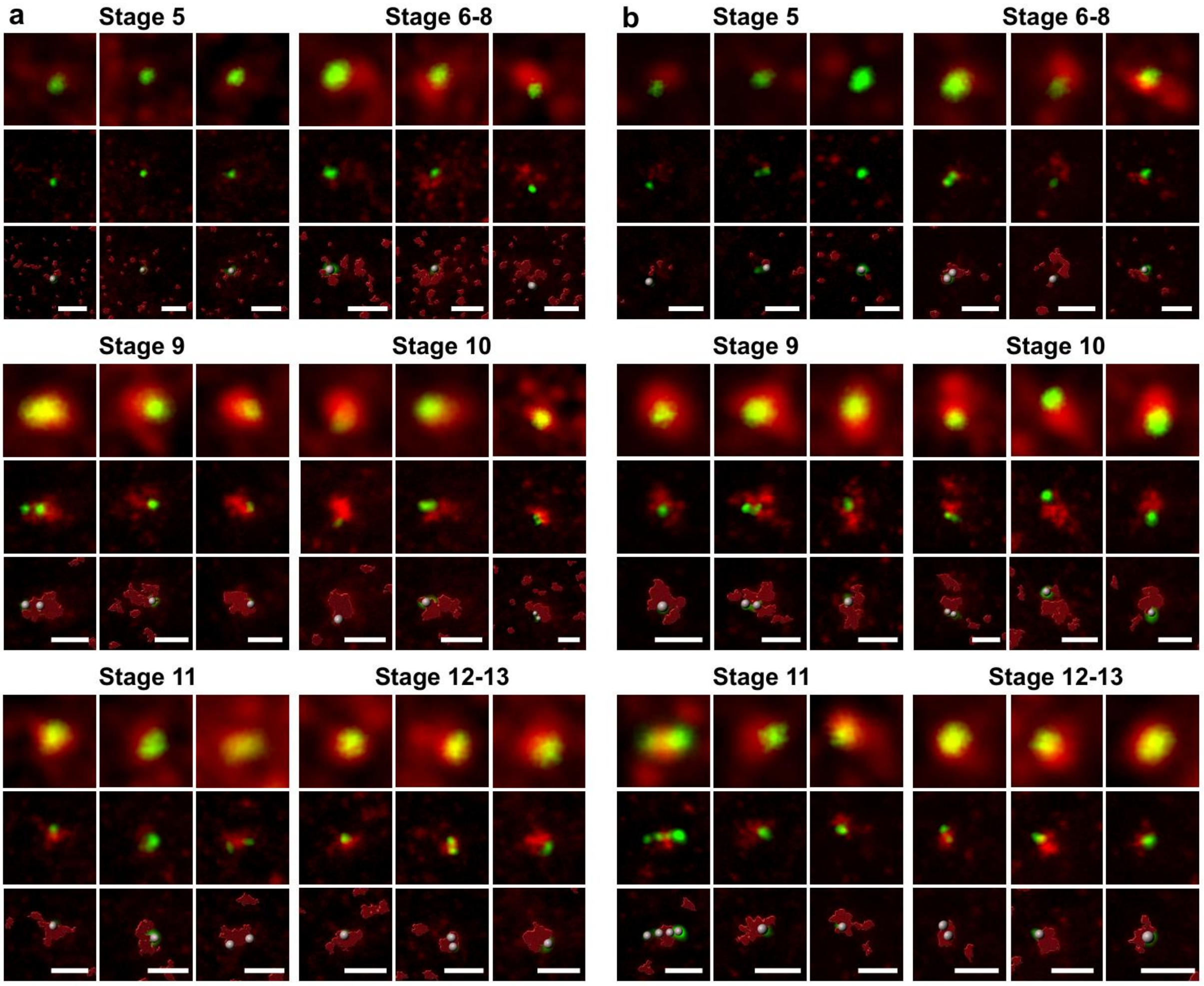

Supplementary Figure 5: Examples of Fab7 localization compared to Ph/Pc substructures during embryogenesis.

a-b: Confocal (first row), STED (second row) and segmented (third row) images of Fab7 (green) compared to Ph (**a**) or Pc (**b**) foci (red) acquired in the head of *Drosophila* embryos illustrating the evolution of Ph/Pc substructures compared to this locus during embryogenesis. A weak Ph or Pc immunostaining signal observed at stage 5 (and stages 6-8 to a lesser extent) explicates the lower association between FISH spots and Ph/Pc substructures during early embryogenesis. Bars measure 500 nm.

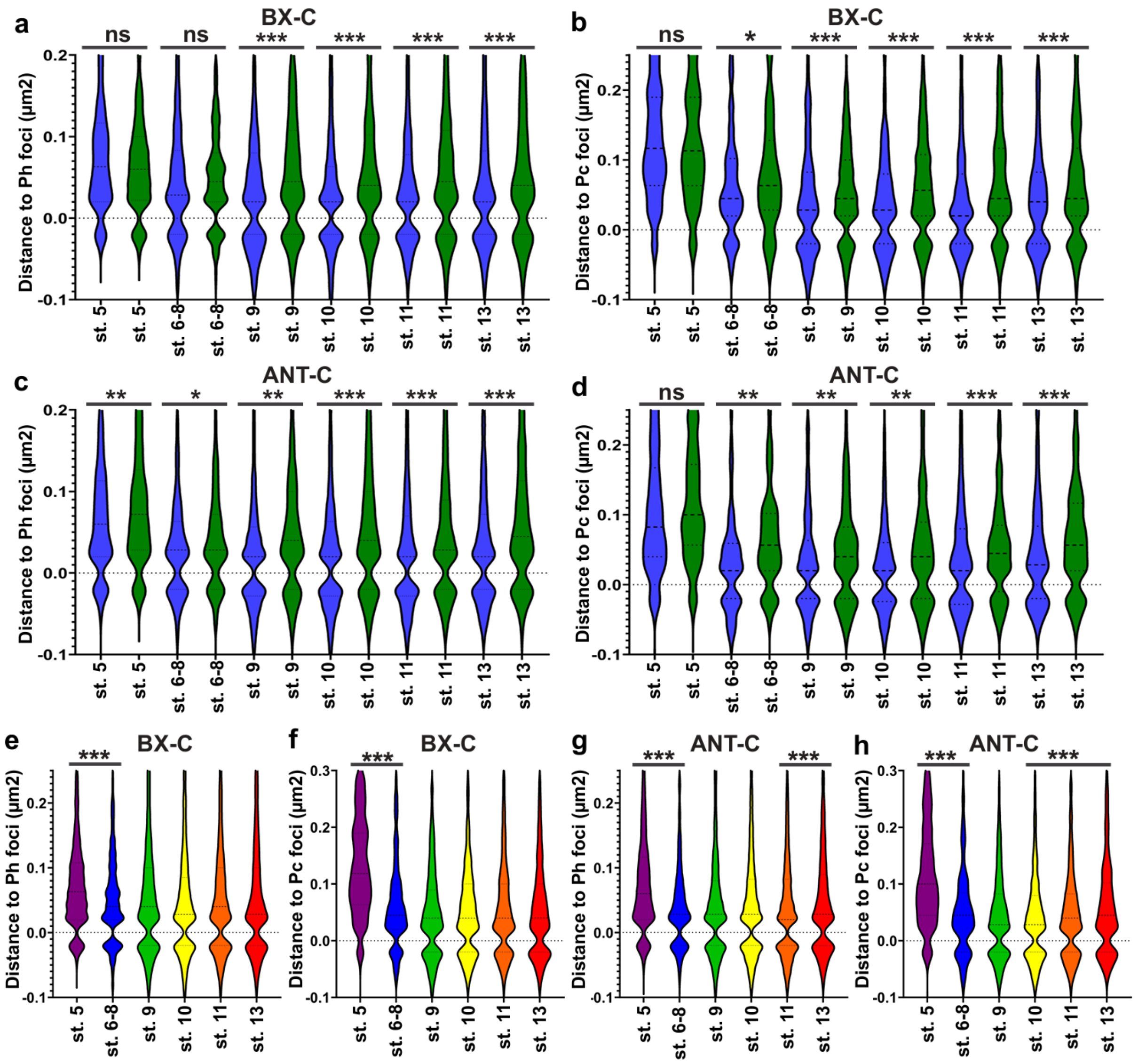

Supplementary Figure 6: Association of Hox chromatin to Ph/Pc substructures during embryogenesis.

a-d: Violin plots showing the distributions of minimum distances measured between the center of FISH spots related to BX-C (**a-b**) or ANT-C (**c-d**) and the border of Ph (**a, c**) or Pc (**b, d**) substructures during embryogenesis. Comparison between PREs located in the middle of Hox clusters (dark blue: bxd and Fab7 for BX-C; Antp and Scr for ANT-C) and intermediate regions (dark green: int1 and int2 for BX-C; int3 and int4 for ANT-C) at embryonic stages 5, 6-8, 9, 10, 11 and 13. Except during Ph/Pc foci formation between stages 5 and 8 when Ph/Pc immunostaining intensity is low (see images of stages 5-8 in Fig. S5), PREs are always more associated with Ph/Pc substructures than intermediate regions. ns: non-significant; *: $p<0.05$; **: $p<0.01$; ***: $p<0.0001$. **e-h:** Violin plots showing the distribution of minimum distances measured between the center of FISH spots and the border of Ph (**e, g**) or Pc (**f, h**) substructures. For simplicity, results from all probes belonging to BX-C (**e-f**), or ANT-C (**g-h**) were merged because PREs and intermediate regions follow similar behavior during embryogenesis. Association of probes located in both BX-C and ANT-C to Ph/Pc substructures robustly increases during formation of Ph/Pc foci (stages 5 to 6-8). During later embryogenesis, BX-C and ANT-C chromatin remains stably associated with Ph/Pc substructures, except for a slight decrease in the association between ANT-C chromatin and Ph/Pc substructures in late embryogenesis (stage 13). ns: non-significant; *: $p<0.05$; **: $p<0.01$; ***: $p<0.0001$.

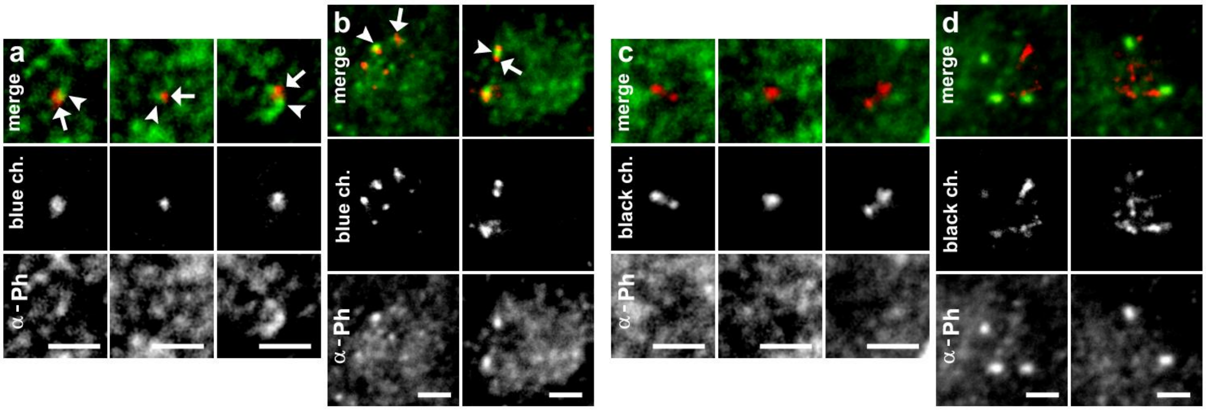

Supplementary Figure 7: Localization of Polycomb-associated chromatin and void chromatin relative to Ph substructures.

2D images of oligopaint probes detecting a single whole genomic domain (**a, c**) or several ones (**b, d**) acquired in the head of embryos by STED microscopy. Images of Polycomb-associated chromatin (**a-b**) show overlap with images of Ph substructures (arrowheads in **a-b**). Similarly to BX-C, the colocalization between Polycomb-associated domains and Ph substructures is only partial (the arrows in **a-b** point regions of the chromatin domains not covered by Ph immuno-labeling). In contrast, void chromatin (**c-d**) is located in nuclear space devoid of Ph substructures. Bars measure 1µm.

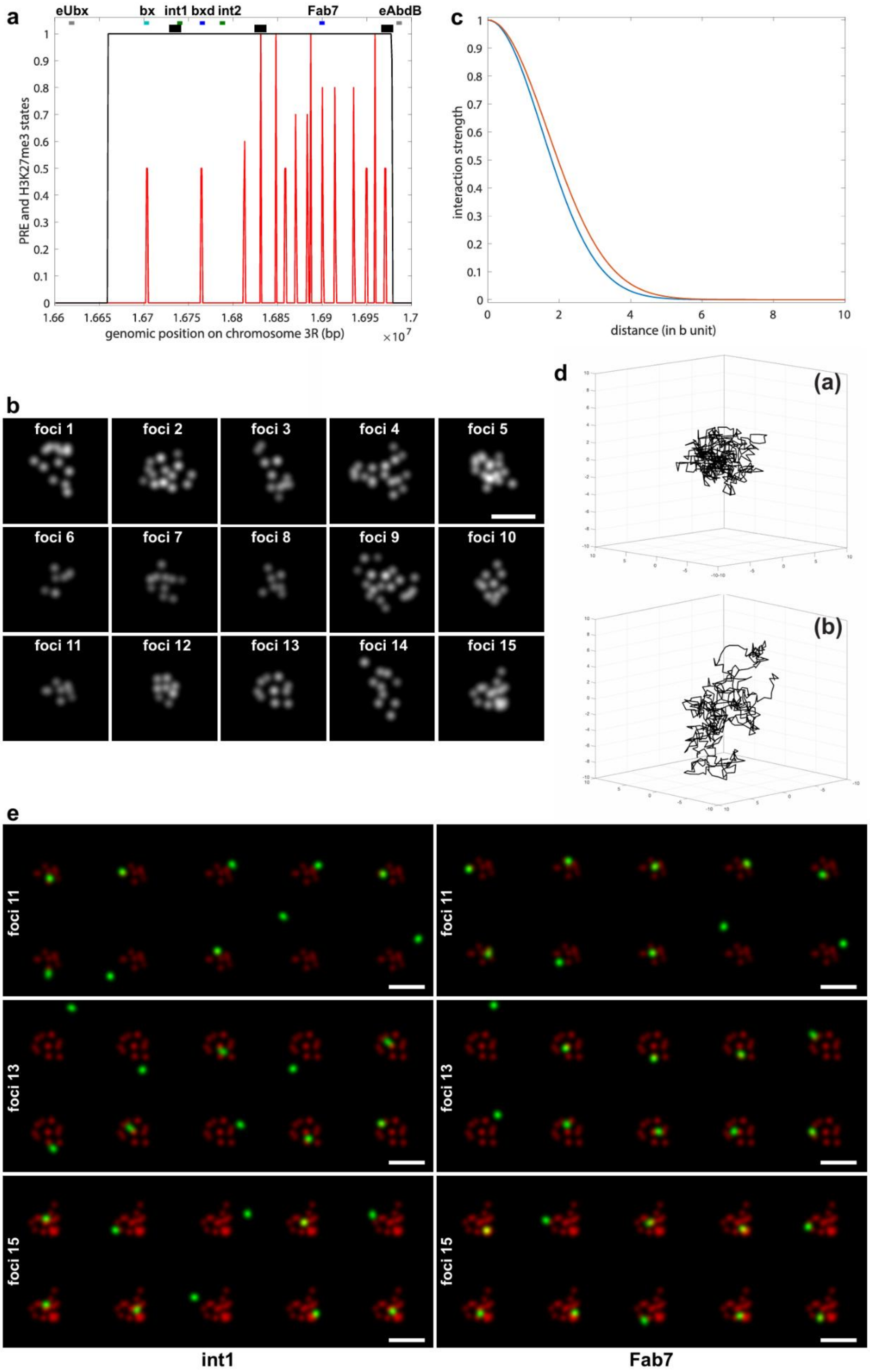

Supplementary Figure 8: Combining polymer simulations and PRC1 nanoglobules to address BX-C's compaction.

a: PRE (p_i, red line) and H3K27me3 (h_i, black line) state for the 400kb-long BX region investigated by simulations at 1kbp resolution. On the top, the 7 rectangles (*top*) represent the 7 probes used to quantify the localization of PRE (blue), H3K27me3 (green) and external (grey) regions and the 3 black rectangles

represent the 3 FISH probes used to measure the compaction of the domain. **b:** Artificial images of the 15 large Ph foci used in the simulations to test the effect of PRC1 nanoglobules on BX-C's compaction. They were calculated using a 70 nm diameter gaussian p.s.f. and the nanoglobules coordinates and intensities measured in 15 large Ph foci (see Fig. 1). Bar measures 500 nm. **c:** Interaction potentials due to excluded-volume $f_{ev}(r)$ (blue line) and attraction $f_{int}(r)$ (red line) between one monomer and one nanoglobule as a function of the distance between the nanoglobule center and the monomer position. Mathematically, these potentials represent the overlap between two 3D gaussian densities: one centered at the nanoglobule position and of radius $\sigma_{NG} = 35$ nm, modeling the nanoglobule; another centered at the monomer position and of radius $\sigma_{ev} = 10$ nm for $f_{ev}(r)$ and $\sigma_{int} = 20$ nm for $f_{int}(r)$. Leading to (with $xx \in \{ev, int\}$):

$$f_{xx}(r) = e^{-\frac{r^2}{[2({\sigma_{NG}}^2+{\sigma_{xx}}^2)]}}/({\sigma_{NG}}^2 + {\sigma_{xx}}^2)^{3/2}$$

d: Examples of simulated configurations for a toy system with just one single nanoglobule centered at position $(x, y, z) = (0{,}0{,}0)$ and a polymer with N=500 monomers with no PRE ($p_i = 0$ for all i) and fully covered with H3K27me3 ($h_i = 1$ for all i) for (J_{ev}, J_h)=(0, 0.8) (a) and (J_{ev}, J_h)=(0.3, 0.8) (b). **e:** Examples of artificial images calculated with the parameter set (J_{ev}, J_h, J_p)=(0.3, 0.4, 5.5) and showing 10 configurations of the loci int1 or Fab7 (in green) relative to nanoglobules (in red) of 3 Ph foci (foci 11, 13 and 15).

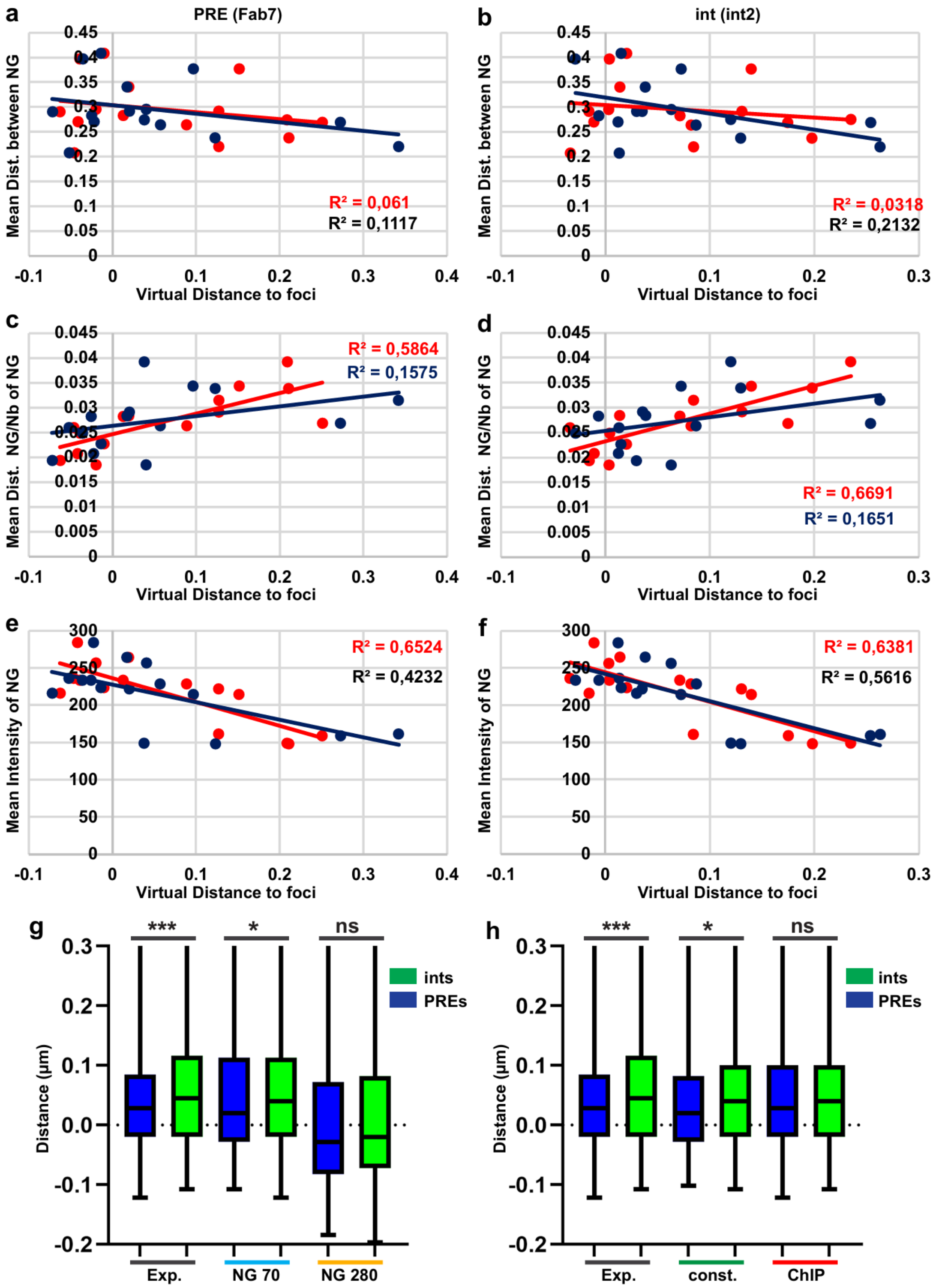

Supplementary Figure 9: The density, intensity and size of PRC1 nanoglobules control the compaction of BX-C.

a-f: Scatterplots comparing the mean distance between PRC1 nanoglobules (**a-b**), PRC1 nanoglobule density (**c-d**) (mean distance between nanoglobules / number of nanoglobules), and the mean Ph labeling intensity within nanoglobules (**e-f**), with the minimum virtual distance measured between Fab7 (**a, c, e**) or int2 (**b, d, f**) loci and the border of Ph nanoglobules. Each point corresponds to a large Ph focus with simulations calculated using the parameters Jev=0, Jh=0, Jp=6.3 (blue); or Jev=0.3, Jh=0.4, Jp=5.5 (red). The intensity of Ph labeling in nanoglobules (**e-f**) or their density (**c-d**) are correlated with BX-C compaction, while weak or

no correlation is observed between the size of large foci (i.e. mean distance between nanoglobules) and BX-C compaction (**a-b**). **g:** Box plot comparing the minimum distances of PREs and intermediate regions to the border of Ph substructures in experimental data (exp.), simulations calculated with Jev=0, Jh=0, Jp=6.3 and 70 nm (NG 70) or 280 nm (NG 280) nanoglobules. ns: non-significant; *: $p<0.05$; ***: $p<0.0001$. **h:** Box plot comparing the minimum distances of PREs and intermediate regions to the border of Ph substructures in experimental data (exp.), simulations calculated with Jev=0.3, Jh=0.4, Jp=5.5 and PREs all having the same intensity (const.) or an intensity modulated according to the Ph ChIP profile (ChIP). ns: non-significant; *: $p<0.05$; ***: $p<0.0001$.

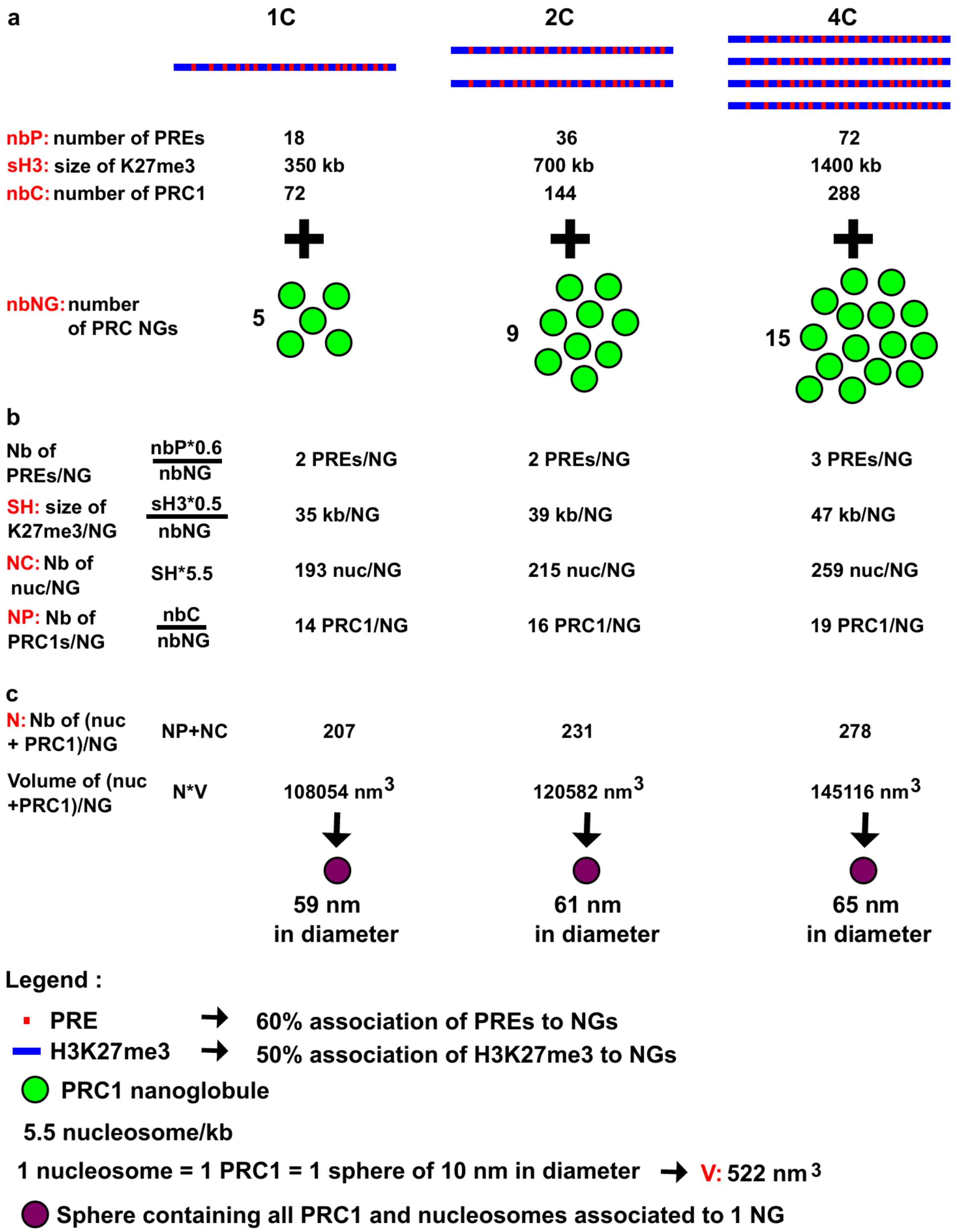

Supplementary Figure 10: Estimation of the volume occupied by the chromatin associated with a PRC1 nanoglobule.

a: Cartoon representing the Hox chromatin associated with 3 large Ph foci containing 5, 9 and 15 nanoglobules and containing 1C (one Hox cluster), 2C (one Hox cluster after replication or during homologous chromosome pairing), or 4C (one replicated and paired Hox cluster), respectively. Each copy of the Hox clusters contains about 18 strong PREs (see **Fig. 2a**) and approximately measures 350 kb. Furthermore, considering that the nucleus of an embryonic

cell contains approximately 2000 PRC1 complexes per C[2] and that a Hox cluster represent about 3.6% of the Polycomb-associated chromatin, approximately 72 PRC1 complexes can bind to one copy of Hox clusters. **b:** Calculation of the number of PREs, the size of H3K27me3 chromatin, the number of PRC1 complexes and the number of nucleosomes associated with a PRC1 nanoglobule. PREs associate with a probability of 60% with PRC1 nanoglobules, whereas the association of intermediate regions with PRC1 nanoglobules occurs with a frequency of about 50% (see **Fig. 2**). In *Drosophila*, 1 kb contains about 5.5 nucleosomes[3]. These estimations are consistent with the previously reported PRC1-dependent compaction of Hox clusters because they indicate that several PREs (2 or 3) are associated to one PRC1 nanoglobule. **c:** Estimation of the total volume occupied by the chromatin associated with a PRC1 nanoglobule and of the diameter of a sphere having the same volume. To simplify the calculation, we assumed that a nucleosome or a PRC1 complex is a sphere with a diameter of 10 nm, which gives a volume of 522 nm^3 per nucleosome or PRC1. Remarkably, for the 3 PRC1 foci we tested, the diameter of a single sphere with the same volume as the total volume of the PRC1 complexes and nucleosomes associated with a nanoglobule is close to the size of the nanoglobules measured by STED microscopy. However, the estimated percentage of nuclear volume occupied by chromatin is below 10 %[4,5], indicating that the volume of a nanoglobule cannot be entirely occupied by the PRC1 complexes and nucleosomes. Overall, estimating the volume occupied by PRC1 complexes and nucleosomes associated with a nanoglobule implies that a large fraction of the chromatin must be located outside the nanoglobule, which is consistent with our observations that Hox chromatin is preferentially enriched near the border of PRC1 substructures, as well as with the partial overlap between Ph and BX-C nanoglobules.

Bibliography Supplementary Figures:

1. Loubiere, V., Papadopoulos, G. L., Szabo, Q., Martinez, A.-M. & Cavalli, G. Widespread activation of developmental gene expression characterized by PRC1-dependent chromatin looping. *Sci. Adv.* **6**, eaax4001 (2020).

2. Bonnet, J. *et al.* Quantification of Proteins and Histone Marks in Drosophila Embryos Reveals Stoichiometric Relationships Impacting Chromatin Regulation. *Dev. Cell* **51**, 632-644.e6 (2019).

3. Baldi, S. *et al.* Genome-wide Rules of Nucleosome Phasing in Drosophila. *Mol. Cell* **72**, 661-672.e4 (2018).

4. Boettiger, A. N. *et al.* Super-resolution imaging reveals distinct chromatin folding for different epigenetic states. *Nature* **529**, 418–422 (2016).

5. Gelléri, M. *et al.* True-to-scale DNA-density maps correlate with major accessibility differences between active and inactive chromatin. *Cell Rep.* **42**, 112567 (2023).

Tables of N value:

Fig. 2:

	Ph Immuno-FISH	Pc Immuno-FISH
	Stages 9-13	Stages 9-13
bxd	1518	920
Fab7	1519	641
Antp	2164	915
Scr	2346	959
bx	1757	876
dAntp	1970	604
int1	1527	862
int2	1539	664
int3	1605	443
int4	1945	991

Fig. 3:

	Distance		Surface And Intensity	
	WT	PcXT109	WT	PcXT109
bxd	394	512	326	450
Fab7	374	531	326	480
Antp	467	522	387	440
Scr	581	588	464	505
bx	374	391	288	329
dAntp	620	680	510	575
int1	451	601	387	495
int2	489	589	384	482
int3	337	385	254	304
int4	630	857	510	644

Fig. 4:

Nb of foci	before9	stade9	early11	stade11	stade12_13
Ph	100	138	134	117	116
Pc	40	94	104	101	97

Supplementary Fig. S2:

	Ph I_Fish	Pc I_Fish
	Stages 9-13	Stages 9-13
bxd	1729	1034
Fab7	1810	788

Antp	2457	1110
Scr	2814	1124
bx	2182	975
dAntp	2328	804
int1	1834	981
int2	1868	813
int3	1952	641
int4	2374	1152
eUbx	398	736
eAbdB	520	817
elab	366	416
cont1	309	NA
cont2	221	NA

Supplementary Fig. S3:

	Ph I_Fish	Ph I_Fish	Pc I_Fish	Pc I_Fish
	Stages 9-13	Stages 9-13	Stages 9-13	Stages 9-13
	Repressed	Expressed	Repressed	Expressed
bxd	1729	562	1034	429
Fab7	1810	848	788	320
bx	2182	824	975	464
int1	1834	907	981	592
int2	1868	916	813	337
Antp	2457	1067	1110	367
Scr	2814	639	1124	288
dAntp	2328	858	804	413
int3	1952	951	641	216
int4	2374	1067	1152	554

Supplementary Fig. S6:

	Dist Ph foci				Dist Pc foci			
	BX-C		ANT-C		BX-C		ANT-C	
	PREs	ints	PREs	ints	PREs	ints	PREs	ints
stage 5	457	468	501	500	260	251	221	231
stage6-8	366	328	317	342	185	95	184	162
stage9	730	832	1040	885	445	397	580	425
stage10	843	849	1305	1204	501	411	513	467
stage11	1040	1156	1760	1252	525	569	671	492
stage 13	735	632	936	745	287	335	385	344

	Dist Ph foci	Dist Pc foci	Dist Ph foci	Dist Pc foci
	BX-C		ANT-C	
stage 5	1302	688	1286	550
stage6-8	943	403	771	375
stage9	2094	1095	2367	1200
stage10	2199	1125	3278	1212
stage11	2836	1381	3639	1376
stage 13	1695	816	2032	852

Supplementary Table S1. List of primers used to produce DNA FISH probes

	name	sens	anti-sens	genomic position	genomic position
**PREs**	bx _frag1	ATCTCGCCATTCTCCGTTCT	ACCACGACTGCTAACTTCCA	16700036	16701625
	bx _frag2	ATTATCACTTCTGGCAGGGC	AAATTGTGCAGCCCTCGTAA	16702038	16703676
	bx _frag3	TAGGCTGCGAAGAGAAGGAG	TGTGTTTGCTCGCATTTCCA	16704046	16705661
	bxd_frag1	TTACCGCGAGAGTTGTGAGT	AAAAGAAGAAGAAGCGGCGG	16762746	16764402
	bxd_frag2	TTTTAGTGGCCTTGCGGTGA	TTCACTTCGACGCGTTTGAC	16764589	16766293
	bxd_frag3	CCGAACGTGGCCACAATAAA	TTCAACCCAGAGTGCTCCAT	16766627	16768281
	Fab7_frag1	GGCATGTAAACCAGCAGAGC	GATTGCCGTTCCATTCGTCA	16896724	16898361
	Fab7_frag2	CCCATTGGTGCAGACTTTGT	TTGGGTTCGGTAAGAGGTCT	16898613	16900258
	Fab7_frag3	AGGCGATCAGGGAACACTTT	TAAGAACGGGCAGCATAGGA	16900719	16902352
	dAntp_frag1	TGTCCGTTTGTCTGTCCGTA	AGTGGCGGCGTAAAGAAATC	7032521	7034174
	dAntp_frag2	TGTGGAGTTTTGTGTTCGCG	ACGGATTTGAGAACCAGGGA	7034674	7036342
	dAntp_frag3	ACCTGTAACCTGTGTCGTGT	CACTAGAAAAGGCGTGGTCA	7036809	7038481
	Antp_frag1	AGCCCTGGCACTTACCTCTT	AGCCGGTCAACTCAAGTCAG	6998210	6999573
	Antp_frag2	CTCGCTGAGCTGCAAACTG	TTTCAGCACCACTGTTCCAC	6999628	7001259
	Antp_frag3	ATCATTCAATTCGGGTCCAG	GGTGAAGGGGCGTAGTGTAA	7002276	7003802
	Scr_frag1	CGGCCAATAGATCAACGACT	GTCTAGCCAGTTTGCCCTCA	6846535	6848089
	Scr_frag2	GATTTGGACGGCTTTTGGTA	TGGCTCAAACGTGAATTGAA	6848201	6849960
	Scr_frag3	GTGGCACTGTTCCGCTAAAT	TGCGTGAAGAACTCAAATGG	6850390	6852075
**Intermediate**	int1_frag1	ATTTCGGCTGTGTCTTTCGG	CGGTCGGCTCAATTTTACGA	16737398	16739084
	int1_frag2	TGTGTGCAAGTGTCGTCAAA	TGCTTTATGGCCAGAATCGC	16739305	16740990
	int1_frag3	TAAGGGTCATTTGCTCCGGT	TCGTTGTGGGTGGTTCTGAA	16741340	16743027
	int2_frag1	AGGCCAAAAGATAGGTCCGT	GAAACCTCTTGACAGCGCAA	16785088	16786711
	int2_frag2	AGCCATCCAATCCGAAATGC	CTTCGAACACTACGCCCATG	16787200	16788890
	int2_frag3	CTCTGAGATTTCTGCGTGGC	ACAAATACCCAGGACCAGCC	16789100	16790830
	int3_frag1	ATACGCGGAAGGATATGGCA	TTTGTGGCAGTGTGAGTGTG	7016161	7017942
	int3_frag2	GCATAAGGAGGCGGTAGTCA	GAATGGCTCGTGGATTGTGG	7018178	7019846
	int3_frag3	AGATTCGGTTTGGGATTCGC	GTACAATATGGTCGGCTGGC	7020104	7021673
	int4_frag1	ATCCGCATCCACATCCTCAT	ATTGCAGCCATGTCGATTCC	6949915	6951585
	int4_frag2	TTGTGTCTGTGCCATTTCGC	CTCGACCAGTGCGTCTTTTG	6951818	6953427
	int4_frag3	CTTTCCCCTCCCCTTCTCAG	AGGTGAACTCAGGGCAGAAA	6953653	6955302
**Adjacent**	e-Ubx_frag1	GAGTGACGAGCTACTGGACA	CTGCCATCTCTTGCGTGAAA	16616022	16617650
	e-Ubx_frag2	AAAAGGTACCCCACTCCTCG	ACCGTTGAGTCTGTTGAGGA	16618052	16619738
	e-Ubx_frag3	AGGCTTCCGTTCGTTGAAAA	AGCTAAGGTGTAGATGGCGG	16620152	16621977
	e-AbdB_frag1	TTAGCCATCGTTGCCCAATG	AGGTTCTTAACTGGCCGGAA	16983104	16984984
	e-AbdB_frag2	CAATCACGCTGGACACTTTG	TTTACCTCCCCGTGAATTTG	16985128	16986819
	e-AbdB_frag3	CCCTCATTTGATGGCGAAGA	GCGTTCAGAGGTCGATGTTC	16987060	16988951
	e-lab_frag1	CCGCCTCAGAAAATTCAGCA	GATTTCCTTGCTCGCCCAAA	6632112	6633723
	e-lab_frag2	TTTCTTTGTCTTTGGCCCGG	CCAGGAATGCAAGGGAATCG	6634225	6635831
	e-lab_frag3	TCCTTAGTTCCCCTTGGCAG	GCCGCGGGATGATATTGTTT	6636288	6637915
**controls**	cont1_frag1	GAATAAAGCGCCGTGACCTT	CAGCATCAACACACATCCGA	17301755	17303361
	cont1_frag2	CTCGGACACACGCAAAAGTT	GCCACATATTAGCGAGCCAC	17303424	17305064
	cont1_frag3	ATGTTTCTCCAGCTGCGAGT	AACTGACAGTGAGTGCGAGT	17305428	17307166
	cont2_frag1	ATCTGCCGCATCGAAGGATA	GCGACCCATGCCAATTGTAT	14977118	14978749
	cont2_frag2	AATTTTGCGTCAGGGCTTCA	ATAATTCCAACTCCGCCGTG	14979277	14980943
	cont2_frag3	ACCCGCAACAACAATCAACA	TTTCGTCTGCTTTTCCTGCC	14981087	14982758
